# Ginsenoside 24-OH-PD from red ginseng inhibits acute T-lymphocytic leukaemia by activating the mitochondrial pathway

**DOI:** 10.1371/journal.pone.0285966

**Published:** 2023-05-19

**Authors:** Qingmiao Li, Yongfu Chen, Xiaolin Zhao, Bocheng Lu, Tingli Qu, Li Tang, Qian Zheng

**Affiliations:** 1 Department of Pharmacy, Shanxi Medical University, Taiyuan, Shanxi, China; 2 Shanxi Traditional Chinese Medicine Hospital, Taiyuan, Shanxi, China; Foshan University, CHINA

## Abstract

Ginsenoside 24-hydroxy-ginsengdiol (24-OH-PD), extracted from red ginseng, is a novel diol-type ginsenoside, strongly inhibits the growth of human T-cell acute lymphoblastic leukaemia (T-ALL) CCRF-CEM cells. Our research aimed at investigating the mechanism underlying this inhibition. Cell viability was determined using the cell counting kit-8 (CCK-8) assay, and NOD/SCID mice bearing CCRF-CEM cells were used to verify the therapeutic effect of 24-OH-PD on T-ALL *in vivo*. We equally analysed pathways related to 24-OH-PD in CCRF-CEM cells using RNA-Seq analysis. Cell apoptosis, reactive oxygen species (ROS), mitochondrial membrane potential (ΔΨm), and mitochondrial permeability transition pore (mPTP) levels were detected by flow cytometry. The activity of caspase3 and caspase9 was detected by enzyme activity detection kits. The expression levels of apoptosis-related proteins and mRNA were determined through western blotting and quantitative reverse-transcription PCR assays (qRT-PCR). CCK-8 assay and animal xenograft experiments confirmed that 24-OH-PD significantly inhibited T-ALL in a dose-dependent manner, both *in vivo* and *in vitro*. RNA-Seq results suggest that mitochondria-mediated apoptosis pathway plays an important role in this process. Furthermore, intracellular ROS levels increased, mPTP opened, and ΔΨm decreased following 24-OH-PD treatment. Pretreatment with the antioxidant, NAC, reversed the effects of 24-OH-PD on apoptosis and ROS generation. Moreover, 24-OH-PD treatment increased the expression of Bax and caspase family members, thereby releasing cytochrome c (Cytc) and inducing apoptosis. Our findings showed that, 24-OH-PD induces apoptosis in CCRF-CEM cells by activating the mitochondrial-dependent apoptosis pathway through ROS accumulation. This inhibitory effect implies that 24-OH-PD could be further developed as treatment of T-ALL.

## 1. Introduction

Acute lymphoblastic leukaemia (ALL) is a malignant tumour where B- or T-lineage cells originating from lymphocytes proliferate abnormally in the bone marrow. Among the types of leukaemia, ALL accounts for approximately 30% of all paediatric tumours [1]. T-ALL mainly occurs in children, and chemotherapy is the most effective treatment at present [2]. However, the resistance of chemotherapy drugs reduces the efficacy of drugs, and eventually leads to a high recurrence rate and poor prognosis of T-ALL. It has been generally recognized in recent years that natural phytochemicals are promising alternatives for therapeutic interventions intended to alleviate the side effects and complications in conventional cancer therapy.

Ginsenosides, as the main active substances in ginseng (*Panax quinquefolius L*.), are well known for their multiple pharmacological activities, among which the function of anticancer has attracted wide attention. Red ginseng is formed by stewing ginseng, during which a large number of ginsenosides undergo hydrolysis, conformational inversion and other changes, forming a series of rare saponins with stronger biological activity and greater advantages in absorption and bioavailability compared with the prototype glycosides [3] (eg. PPD [4], 20(S)-25OCH3-PPD [5] and CK [6]). 20 (S)-Dama-20,25-epoxy-3β,12β,24α-triol (24-OH-PD) is a natural diol dammarane ginsenoside isolated and obtained from red ginseng by our research group for the first time. The structure of 24-OH-PD is different from that of ginsenosides which have been reported to have strong anticancer activity. The side chain of 24-OH-PD at position 17 is a hydroxyl oxygen-containing six-membered ring [7], rather than the open chain structure of the protoginsenediol type (such as Rg3, Rh2, PPD, ginsenoside CK). Previous functional studies have shown that 24-OH-PD has inhibitory effects on a variety of tumor cells, witch is much stronger than that of ginsenoside Rg3, and is comparable to that of Rh2 [8] (ginsenoside with strong anticancer activity against cancer cell proliferation [3]). In vivo pharmacokinetic studies showed that it had rapid and extensive distribution, no accumulation, and its pharmacokinetic characteristics were more suitable for patent drugs, and its in vivo half-life (T1/2:4.82± 1.99–5.45 ±1.83 h) is much longer than that of similar compounds [9]. However, the mechanism by which it inhibits tumor cell viability remains unclear.

Thus, we explored the anti-tumour effects of 24-OH-PD both *in vitro* and *in vivo*. We observed that mitochondrial pathways are involved in 24-OH-PD-induced apoptosis of ALL cells. This provides an experimental basis for the use of 24-OH-PD as anti-cancer treatment, thereby laying a theoretical foundation for further development.

## 2. Materials and methods

### 2.1 Cell culture

The CCRF-CEM (CCL-119) cell lines were purchased from Guangzhou Jennio Biotech Co., Ltd (JNO-18249) and were cultured in RPMI-1640 (Gibco, America) with 10% FBS (Gibco, America), supplemented with 100 units/ mL penicillin, and 100 μg/mL streptomycin (Gibco, America). They were incubated at 37°C with 5% CO_2_. 24-OH-PD and 20(S)-ginsenoside Rh2 (20 (S)-GRh2) were provided by the Jilin Ginseng Innovative Drug Development Engineering Research Center, Jilin University, and were detected using high-performance liquid chromatography-evaporative light scattering detection (HPLC-ELSD). The purity was found at 99.87% and 97.69%, respectively. 24-OH-PD was dissolved in dimethyl sulfoxide (DMSO, Beyotime, China) as the stock solution.

### 2.2 Cell viability assay

CCRF-CEM cells were cultured to the logarithmic phase and treated with various concentrations of 24-OH-PD for 24 h. The absorbance at 450 nm was measured after adding CCK-8 assay according to the manufacturer’s instructions (Beyotime, China), and all assays were performed in triplicate.

### 2.3 Animal administration and sample collection

The experimental protocols related to animals were approved by the Experimental Animal Ethics Committee of the Shanxi Medical University (2020ZSLYEC-225 date of approval: 20200309). Thirty NOD/SCID mice (half male and half female, 6–8 weeks old) were purchased from Guangzhou Jennio Biotech Co., Ltd. (SCXK-yue20200054). All animals were fed and allowed to walk freely in wire cages. They were kept in an animal room at constant temperature (25 ± 2°C), humidity (45 ± 10%), and within 12 h dark/light cycle. They were acclimatised for 1 week before the experiments.

NOD/SCID mice were divided into the following groups: (a) vehicle control group (n = 8); (b) 1 mg/kg 24-OH-PD group (n = 10); (c) 10 mg/kg 24-OH-PD group (n = 10); and (d) control group (n = 2). IV with CCRF-CEM cells (1 × 10^7^ cells in 0.2) via the tail vein of mice from groups a, b, and c. The vehicle or 24-OH-PD was injected IP from day 0 onwards, quaque die for five consecutive days as one course. They received six courses in total, and a 2-day break between any two courses. The injected animals were observed daily for signs and symptoms of illness and scored twice weekly based on parameters previously reported by our group [10].

On the 25th day, the mice in the blank group (n = 2), vehicle control group (n = 2), low-dose group (n = 2), and high-dose group (n = 2) were euthanized by CO_2_ overdose inhalation. The liver and spleen were collected and fixed in a 4% formaldehyde solution, after which paraffin was embedded, sectioned in 3 μm portions, haematoxylin and eosin (HE) stained, and histopathological morphological manifestations of the organs were observed under light microscope.

### 2.4 RNA-sequencing

CCRF-CEM cells were treated with various concentrations of 24-OH-PD (0, 60, 70, and 80 μM) for 24 h, and total RNA was extracted using the TRIzol reagent (Takara, Japan). The quality of the extracted samples was evaluated using an Agilent 2100 Bioanalyzer (Agilent Technologies, America). Beijing Novogene Technology Co., Ltd. performed the DNA library construction, library purification, and transcriptome sequencing analysis. The RNA-Seq data were uploaded to the NCBI Sequence Read Archive (SRA) database at PRJNA841242. Findings related to the quality analysis of the sample sequencing data are presented in S1 Table. Using the independent research and development process, differential expression analysis of the two groups was performed using the DESeq2 R package (1.20.0). The resulting P-values were adjusted using Benjamini and Hochberg’s approach to control the false discovery rate. Padj < 0.05 was set as the threshold for significantly differential expression. We used the clusterProfiler R package (3.8.1) to test the statistical enrichment of DEGs in the KEGG pathways and cluster analysis. Protein-protein interaction(PPI) analysis was performed using string.

### 2.5 Apoptosis assay

Apoptosis was detected using an Annexin V-FITC/PI Apoptosis Detection Kit (Beyotime, China). After adding 24-OH-PD and 70 μM Rh2 for 24 h, CCRF-CEM cells were washed twice with pre-cooled PBS (Beyotime, China), resuspended in AnnexinV-FITC/PI composite staining solution, incubated at 37°C for 30 min, and analysed using flow cytometry (FCM: BD Biosciences, USA). In addition, to test the effect of reactive oxygen species, the cells were pretreated with 5 mM N-acetyl cysteine (NAC, Beyotime, China) for 1 h, and the rate of apoptosis was determined as described above.

### 2.6 Caspase-3 and Caspase-9 activity

CCRF-CEM cells incubated with 24-OH-PD for 24hd were detected using Caspase-3 and Caspase-9 active enzyme activity assay kits (Beyotime, China). After lysis of the treated cells, samples, assay buffer, and Caspase-3, -8, or -9 substrates were mixed according to the manufacturer’s instructions in proportions and incubated for 2.5h at 37° C in the dark. The optical density (OD) was determined at 405 nm by a microplate reader and the activity of caspase was presented by calculating the ratio of OD (sample)/OD (negative).

### 2.7 Quantitative reverse-transcription PCR analysis

Total RNA was extracted using TRIzol reagent and reverse-transcribed using the PrimeScript RT kit (Takara, Japan). Amplification was performed using SYBR Premix Ex Taq II (Takara, Japan) and was detected using a PCR amplifier (Thermo Fisher Scientific, America). β-Actin was used as an internal reference to normalise the results. The primer sequences (Sangon Biotech, China) are shown in S2 Table.

### 2.8 Western blotting analysis

Total proteins were extracted using the Nuclear and Cytoplasmic Protein Extraction kit (Beyotime, China) or the Mitochondrial Protein Extraction kit (BOSTER, China), and the protein concentration was determined using the BCA Protein Quantification Kit (BOSTER, China). The proteins were subjected to 10% SDS-PAGE and then transferred to a PVDF membrane, blocked with TBST containing 5% skim milk, and probed with Bax (Abclonal, China), Caspase-3 (Abclonal, China), Caspase-9 (Abclonal, China), and cytochrome c (Cytc, Abclonal, China) monoclonal antibodies. The protein bands were visualised using an enhanced chemiluminescence detection kit (BOSTER, China) and photographed using a gel imager (Bio-Rad, America).

### 2.9 Analysis of mitochondrial membrane potential

The mitochondrial membrane potential assay kit with JC-1 (Beyotime, China) and the Mitochondrial Permeability Transition Pore Assay kit (Beyotime, China) were used to measure mitochondrial membrane potential (ΔΨm) and mitochondrial permeability transition pore (mPTP), respectively, in CCRF-CEM cells. The ΔΨm was quantified by measuring the relative content of mitochondrial JC-1 monomers and aggregates using flow cytometry.

For mPTP detection, cells were stained with calcein AM according to the manufacturer’s instructions and detected using excitation and emission wavelengths of 494 and 517 nm, respectively. All data were analysed using the FlowJo software(10.4).

### 2.10 Measurement of intracellular reactive oxygen species generation

Intracellular ROS production was determined using reactive oxygen species detection kit (Beyotime, China). Cells were harvested, washed twice with RPMI-1640 medium, and incubated with DCFH-DA working solution at 37°C for 30 min. After washing three times with medium, the cells were resuspended in 1 mL PBS for flow cytometry analysis.

CCRF-CEM cells were pretreated with 5 mM NAC for 1 h, and ROS levels were determined using the aforementioned method.

### 2.11 Statistical analysis

All variables are presented as the mean ± standard error (SEM). Data were analysed using GraphPad Prism 9. The independent samples t-test was used to analyse the difference between the means of the two samples, and the one-way ANOVA method for the means of multiple groups. Statistical significance was set at P < 0.05, and all experiments were performed in triplicate.

## 3. Results

### 3.1 24-OH-PD inhibits the proliferation of CCRF-CEM *in vitro and in vivo*

**Fig 1:**
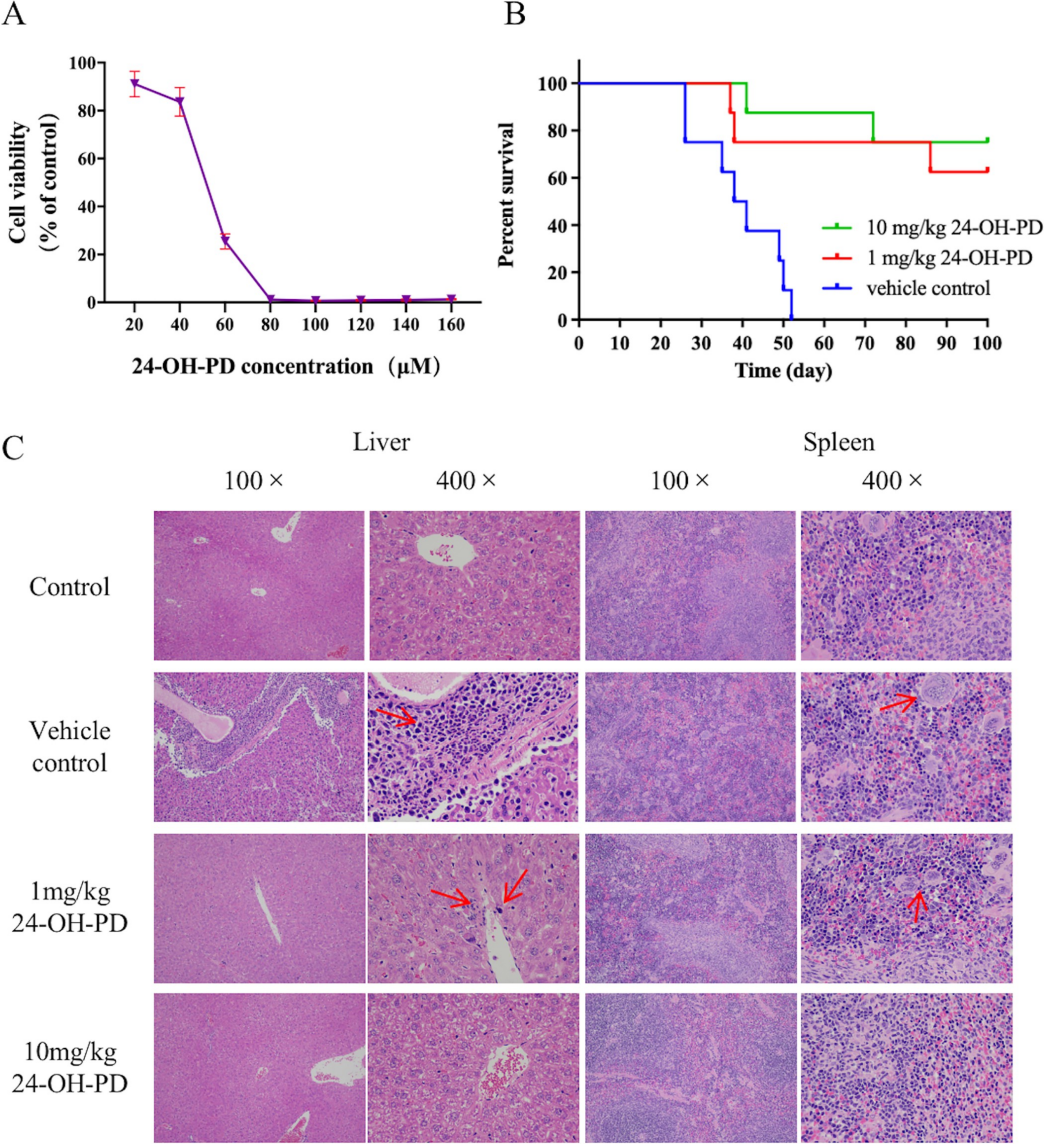
24-OH-PD inhibits the proliferation of CCRF-CEM in vitro and in vivo. **(A)** 24-OH-PD inhibited the viability of CCRF-CEM cells. Cells were treated with different concentrations of 24-OH-PD for 24 h, then measured for cell viability by the CCK-8 assay. Error bars indicate SD. **(B)** The survival curves of the CCRF-CEM cell-bearing NOD/SCID mouse model in the vehicle control group, 1 mg/kg 24-OH-PD group, and 10 mg/kg 24-OH-PD group (n = 8 per group). **(C)** HE-stained spleen and liver pathological sections of mice in different treatment groups.

The effect of 24-OH-PD on the viability of CCRF-CEM cells was assessed using CCK-8 assay. As shown in Fig 1A, 24-OH-PD significantly reduced the viability of CCRF-CEM cells in a dose-dependent manner (IC_50_ = 50.91 μM). Therefore, we used 24-OH-PD at concentrations of 60, 70, and 80 μM for subsequent experiments.

After assessing apoptosis of CCRF-CEM cells *in vitro*, we further investigated the efficacy of 24-OH-PD *in vivo*. The survival curve of the CCRF-CEM cell-bearing NOD/SCID mouse model is shown in Fig 1B. The results showed that 24-OH-PD had a good inhibitory effect on the T-ALL mice; both the high-dose and low-dose groups experienced significant involvement in the survival rate, and the 100-day survival rate was 63% and 85%, respectively. The HE staining showed that 24-OH-PD significantly delayed the course of T-ALL. As shown in Fig 1C, cancer cells and lesions in the liver and spleen of T-ALL mice were significantly smaller than those in the solvent control group. In addition, no significant damage was observed during the long-term use of 24-OH-PD in mice, indicating that the drug was apparently not toxic at 10 mg/kg.

### 3.2 RNA-Seq analysis revealed that 24-OH-PD inhibited T-ALL mainly by inducing apoptosis

**Fig 2:**
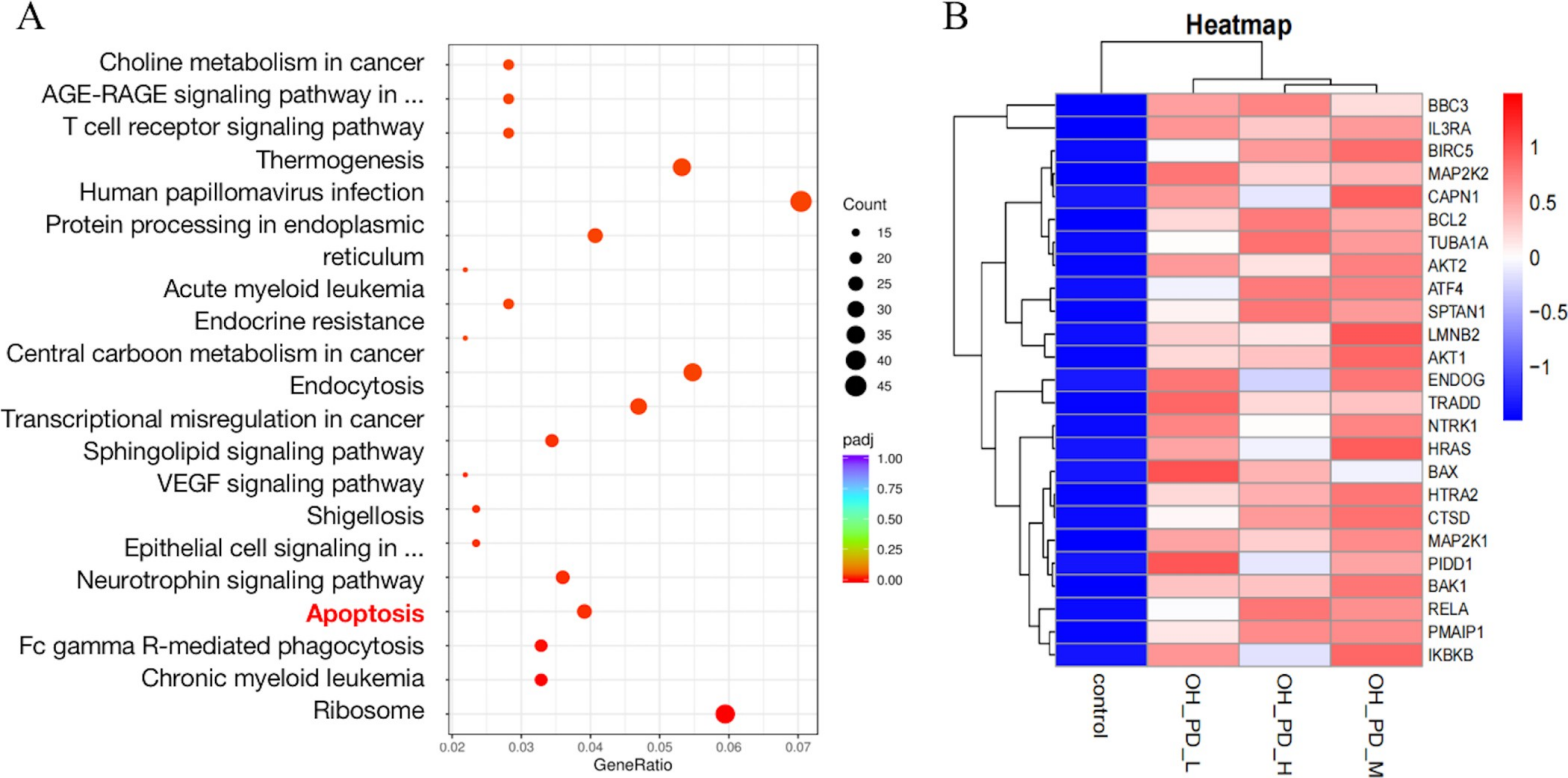
RNA-Seq analysis. (A) Scatter plot of KEGG pathway enrichment for the top 20 significant DEGs. (B) Cluster analysis of up-regulated DEGs from apoptosis pathway.

To explore the molecular mechanism by which 24-OH-PD inhibiting the viability of CCRF-CEM cells, RNA-Seq was used to compare 24-OH-PD-treated and -untreated cell samples. Sequencing was performed using Illumina, and clean reads were obtained after evaluating the sequencing data. We obtained 3,180 differently expressed genes (DEGs) between the treated and untreated samples according to their expression levels (Padj < 0.05). There were 1,694 upregulated and 1,486 downregulated genes in the 24-OH-PD-treated cells. These DEGs were enriched in 43 pathways (Padj < 0.05) and the top 20 pathways are shown in a scatter plot. As shown in Fig 2A, the most prominent pathways involved endocytosis, ribosomes, human papilloma virus infection, apoptosis, and protein processing in the endoplasmic reticulum, indicating that these pathways are closely related to CCRF-CEM cell apoptosis caused by 24-OH-PD. The up-regulated DEGs of the apoptosis pathway were focused, and changes in FPKM values were analysed using cluster analysis (Fig 2B).

### 3.3 Apoptosis induction by 24-OH-PD in CCRF-CEM cells

**Fig 3:**
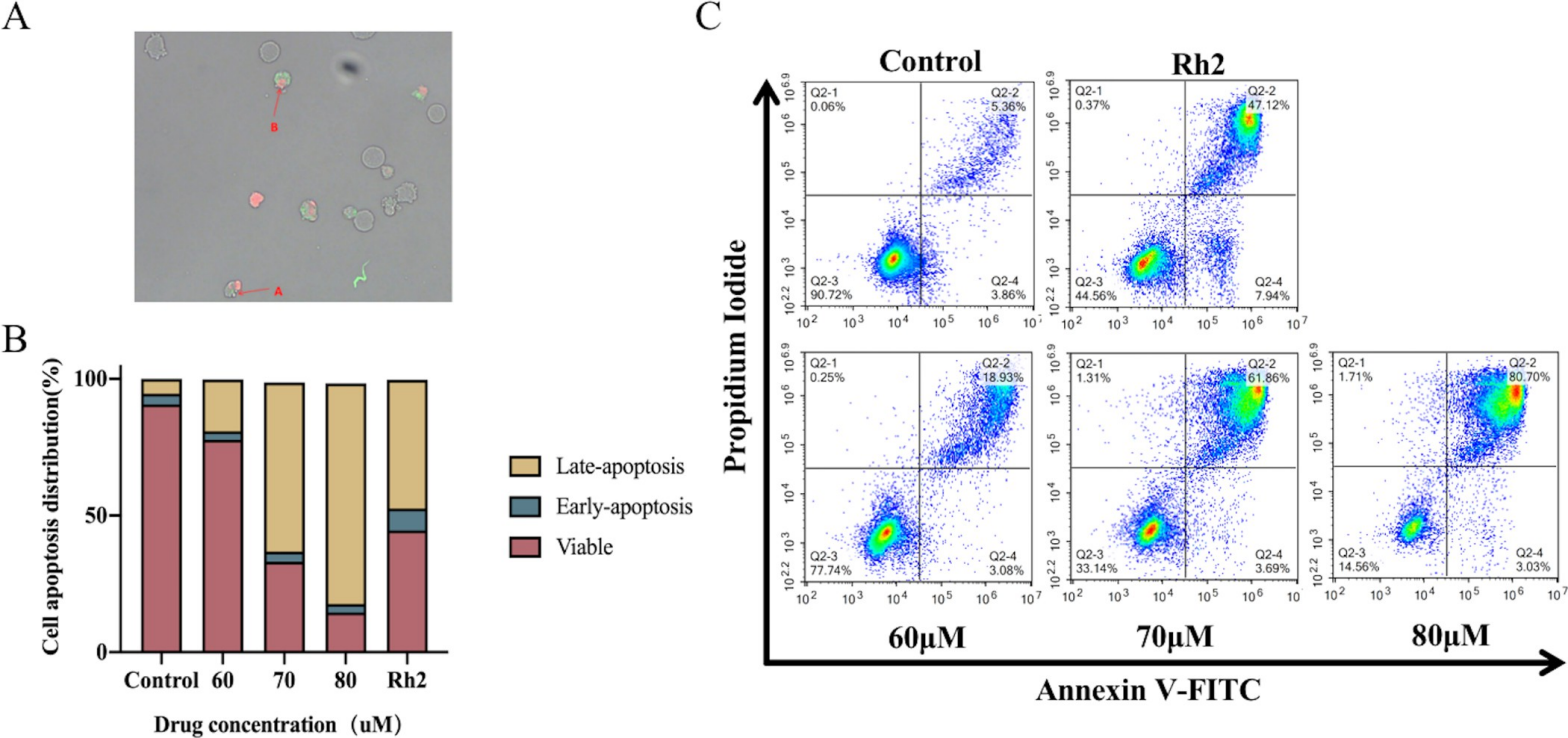
Inhibitory effect of 24-OH-PD on CCRF-CEM cells. (A) Cell morphology of CCRF-CEM cells treated with 80 μM 24-OH-PD for 24 h. A fluorescence microscope was observed at 400× and AnnexinV-FITC and PI staining. (B) Quantitative analysis of apoptosis of CCRF-CEM cells. Data are shown as means ± SD of three separated experiments. (C) CCRF-CEM cells were treated with 24-OH-PD (0, 60, 70, and 80 μM) and 70 μM Rh2 for 24 h after AnnexinV-FITC, PI staining, and then analysed using flow cytometry.

Since the transcriptome results showed that apoptosis may play an important role in the inhibition of cell proliferation by 24-OH-PD, we further examined the cell apoptosis using Annexin V-FITC/PI kit. As shown in Fig 3A, fluorescence microscopy revealed that cells treated with 24-OH-PD (80 μM) showed apoptosis-related features. Arrow A shows cell membrane budding and vesicle formation. Arrow B shows that the nuclei are concentrated and pyknotic or clump-shaped. Flow cytometry results are shown in Fig 3B and 3C. When the concentration of 24-OH-PD increased from 0 to 80 μM, the proportion of apoptotic cells significantly increased from 9.22% to 83.73%, and the cells became apoptotic but did not undergo necrosis. These findings indicated that 24-OH-PD significantly inhibited the proliferation of CCRF-CEM cells through an apoptotic mechanism.

### 3.4 The mitochondrial pathway is activated in 24-OH-PD-induced apoptosis

**Fig 4:**
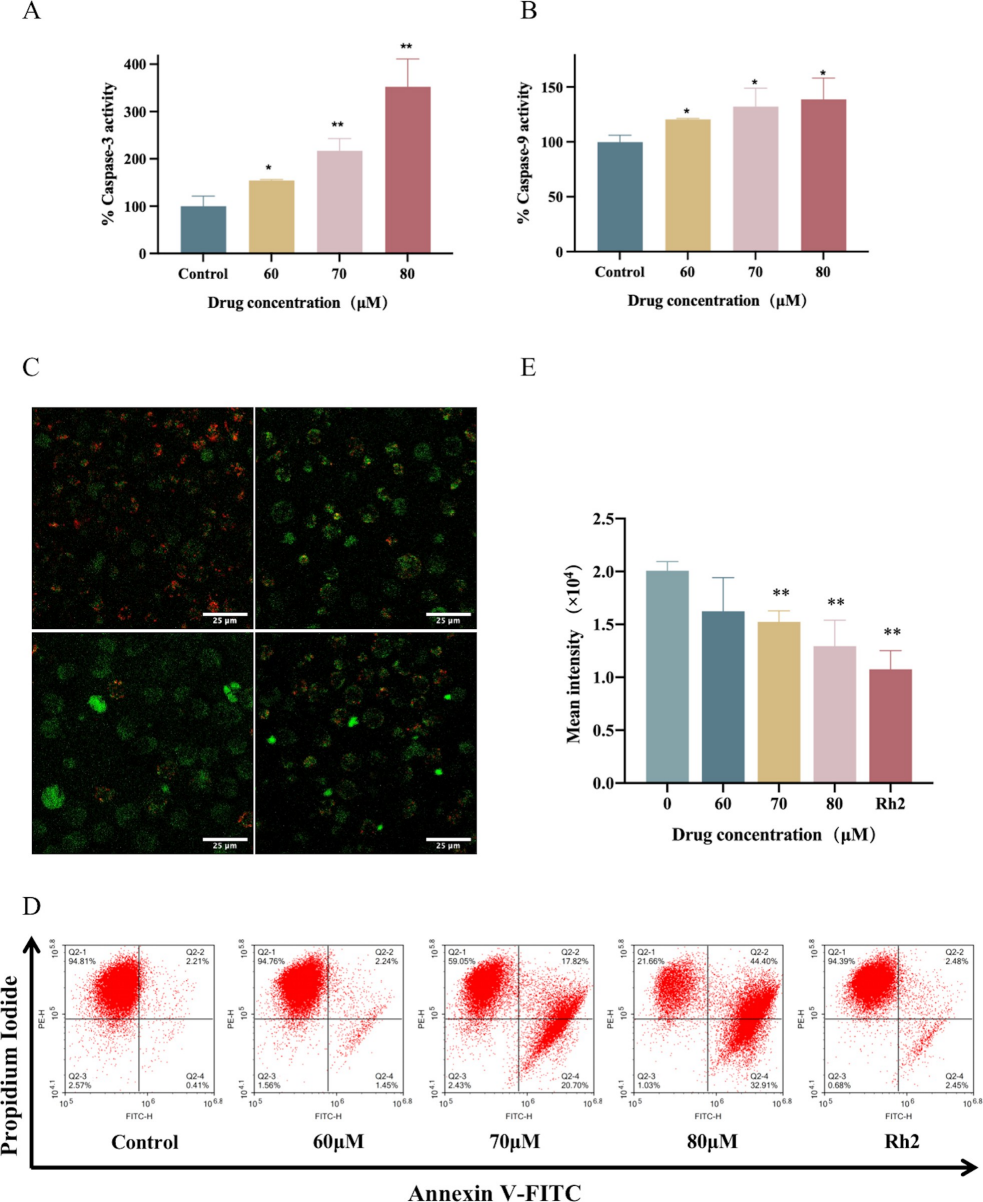
Effect of 24-OH-PD on the mitochondrial membrane potential in CCRF-CEM cells. CCRF-CEM cells were cultured in 24-OH-PD (0, 60, 70, and 80 μM) and 70 μM Rh2 for 24 h. (A) and (B) Effect of 24-OH-PD on caspase 3 and 9 activity in CCRF-CEM cells. Data are shown as means ± SD of three separated experiments. (C) Fluorescence images of mitochondrial staining in CCRF-CEM cells detected using laser scanning confocal microscopy. (Red indicates JC-1 aggregates, and green fluorescence indicates JC-1 monomers. Scale bar = 25 μmol/L). (D) Cell mitochondrial membrane potential was measured using flow cytometry. (E) mPTP was measured using flow cytometry. (*P < 0.05, **P < 0.01).

To explore the mechanism of 24-OH-PD inducing apoptosis, we analyzed the apoptotic up-regulated DEGs obtained by transcriptome. The results showed that a large number of DEGs closely related to mitochondrial pathways were significantly up-regulated, including BAX, AKT1, BAK1, ENDOG, BBC3, PMAIP1, CTSD, PIDD1, and HTRA2 (Fig 2B). In addition, the activity of Caspase enzymes showed that 24-OH-PD could significantly up-regulate the activities of caspase 3 and 9 (Fig 4A and 4B). These results suggest that the mitochondria-mediated apoptotic pathway plays an important role in 24-OH-PD-induced apoptosis.

Mitochondrial depolarisation is an early stage of the mitochondrial apoptosis activation pathway and it often reflects an increased JC-1 polymer/monomer ratio [11]. ΔΨm was stained with JC-1 dye to further verify mitochondrial changes. As shown in Fig 4C, compared to that of the control group, the green fluorescence intensity of the administration group gradually increased with increasing concentration, while that of the red fluorescence gradually decreased. Flow cytometry was performed to quantify the results. As shown in Fig 4D, the ΔΨm of CCRF-CEM cells was significantly depolarised (P < 0.05) after treatment with 24-OH-PD, and the red/green fluorescence intensity decreased with drug concentration in a dose-dependent manner.

The degree of mPTP opening was assessed using flow cytometry. As shown in Fig 4E, the intensity of green fluorescence decreased with increasing treatment concentrations, revealing a higher mPTP openness. In the present study, 24-OH-PD significantly reduced ΔΨm in CCRF-CEM cells and increased the degree of mPTP opening, thereby further activating the mitochondrial apoptotic pathway.

### 3.5 24-OH-PD regulates the expression levels of genes and proteins in mitochondrial pathway

**Fig 5:**
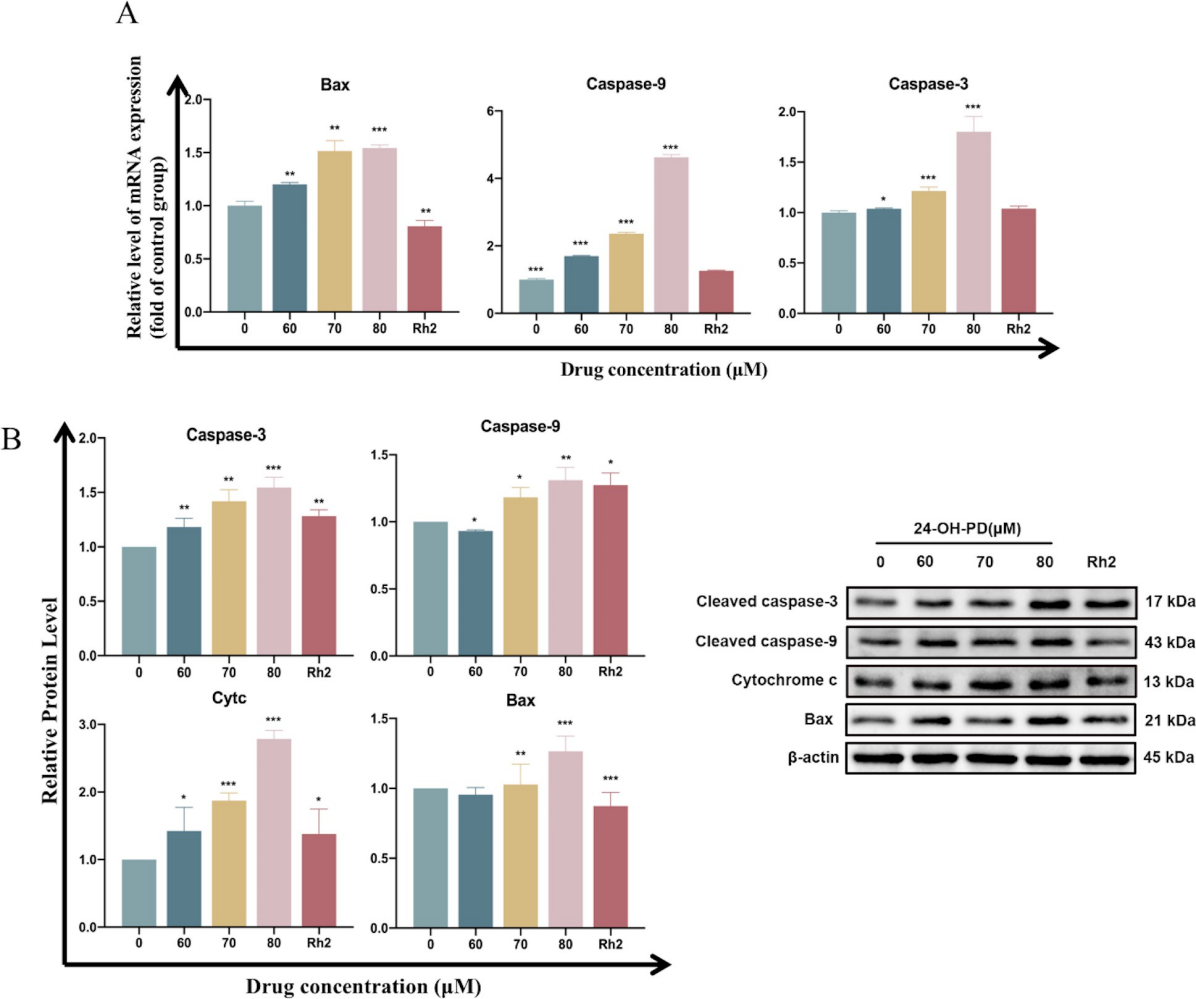
Changes in gene transcription and protein expression in CCRF-CEM cells. CCRF-CEM cells treated with different concentrations of 24-OH-PD (0, 60, 70, and 80 μM) and 70 μM Rh2 for 24 h. (A) The mRNA transcript levels of the Bax, Caspase-3 and Caspase-9 genes. (B) Protein expression levels of Bax, Cytc, Caspase-3 and Caspase-9 (*P < 0.05, **P < 0.01, ***P < 0.001).

In order to monitor the activation of mitochondrial pathways, we examined the expression of apoptosis-related genes using qRT-PCR analysis. As shown in Fig 5A, the mRNA of Bax, Caspase-3 and Caspase-9 were significantly upregulated by 24-OH-PD (*P* < 0.05) in a concentration-dependent manner. Protein expression levels were further analysed through western blotting. As shown in Fig 5B, the expression levels of Bax, Caspase 3 and Caspase-9 were significantly upregulated (P < 0.05) in a dose-dependent manner, consistent with the qRT-PCR results. Moreover, an upregulation of Cytc reveals that 24-OH-PD induces an endogenous mitochondrial apoptosis pathway by regulating the Bax and Cytc.

### 3.6 Role of ROS levels in mitochondrial activation by 24-OH-PD

**Fig 6:**
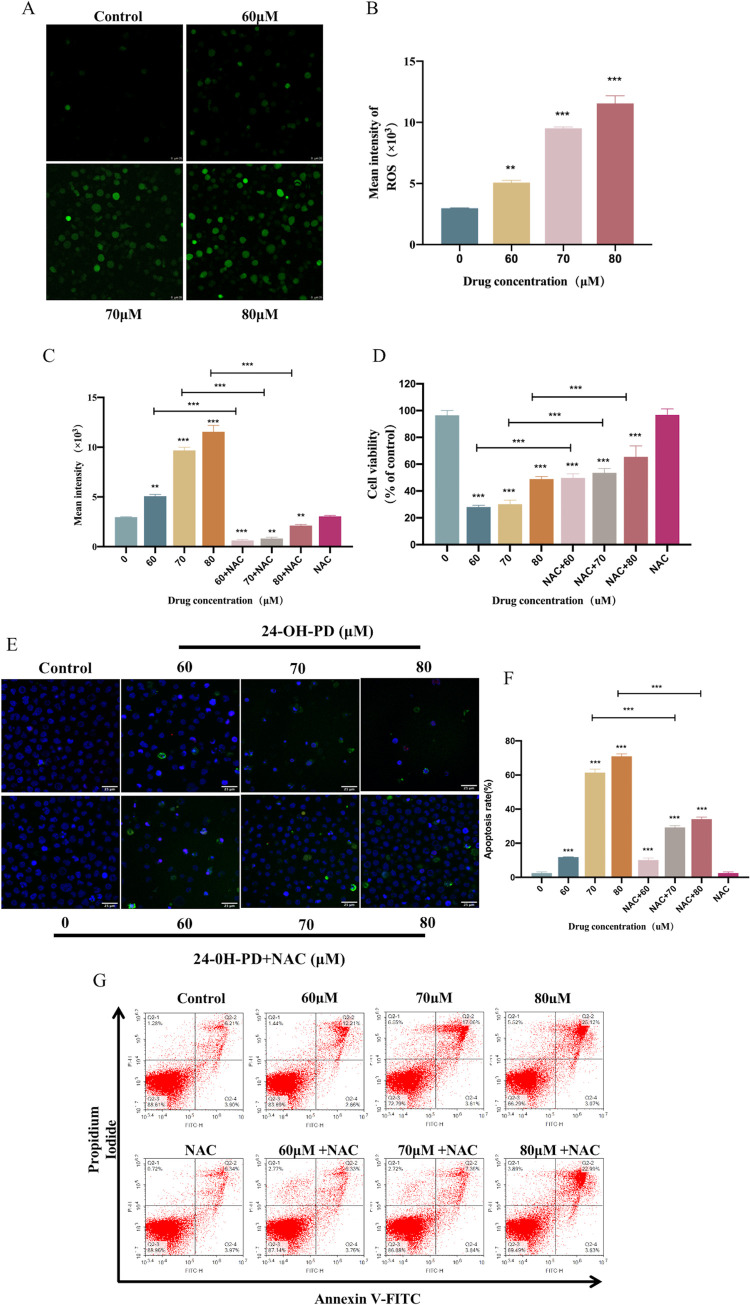
Effects of 24-OH-PD on ROS production in CCRF-CEM cells. CCRF-CEM cells were treated with 24-OH-PD (0, 60, 70, and 80 μM) for 24 h, and the DCFH-DA probe was used to stain ROS. (A) Fluorescent images of mitochondrial staining were detected using laser scanning confocal microscopy. Scale bar = 25 μmol/L. (B) Cell ROS levels were measured using flow cytometry. (C) and (D) CCRF-CEM cells were pretreated with NAC (5 mM) for 1 h, then treated with 24-OH-PD (60, 70, and 80 μM) for 24 h. ROS accumulation and cell viability was measured using the DCHF-DA probe and CCK-8 assay, respectively. (E) and (G) CCRF-CEM cells were first pretreated with NAC (5 mM) for 1 h and then treated with 24-OH-PD (60, 70, and 80 μM) for 24 h and analysed for apoptosis using laser scanning confocal microscopy and flow cytometry, respectively.Scale bar = 25 μmol/L. (F) Quantitative analysis of apoptosis of CCRF-CEM cells. Data are shown as means ± SD of three separated experiments. (*P < 0.05, **P < 0.01, ***P < 0.001).

The aforementioned studies have revealed that 24-OH-PD-related apoptotic induction in CCRF-CEM cells is related to the mitochondria. Moreover, oxygen radicals from the mitochondria are closely related to the pathogenesis of cancer. To further explore whether 24-OH-PD-related apoptotic induction was related to ROS, we analysed fluorescence expression using DCFH-DA-labelled ROS. As shown in Fig 6A and 6B, 24-OH-PD increased ROS production in CCRF-CEM cells in a dose-dependent manner. Excessive ROS produces cytotoxicity, indicating that oxidative stress is an essential feature in 24-OH-PD-induced apoptosis.

To verify the critical role of ROS in 24-OH-PD-induced apoptosis, we pretreated cells with the conventional antioxidant NAC and retested ROS, cell viability and apoptosis. As shown in Fig 6C and 6D, NAC inhibits the production of ROS and cell viability in 24-OH-PD-treated cells in a concentration-dependent manner. Furthermore, laser scanning confocal microscopy and flow cytometry results indicated that NAC could significantly reverse apoptosis (Fig 6E–6G). These confirmed that 24-OH-PD-related apoptotic induction was ROS-dependent.

## 4. Discussion

We explored gene expression changes in the system using RNA-Seq analysis, and identified 3,180 DEGs in 24-OH-PD-treated cells. KEGG pathway enrichment analysis of the DEGs data revealed that some of pathways were those regulating apoptosis, such as endocytosis, ribosomes, apoptosis, and protein processing in the endoplasmic reticulum pathway. Then, cluster analyses and PPI analyses were performed on the up- regulated DEGs of apoptotic pathway. The results showed that among the core genes regulated by 24-OH-PD, a large number of genes were enriched in the mitochondria-mediated apoptosis pathway, such as Bax, BAK1, ENDOG, BBC3, PMAIP1 and HTRA2 [12–17]. The mitochondrial pathway is one of the most common pathways of ginsenoside-induced apoptosis in tumour cells [18]. As the accuracy of the transcriptome results requires further experimental verification [19], and in order to study how 24-OH-PD activates the mitochondrial pathway, we targeted the key genes in the mitochondrial pathway for detailed verification.

Caspases, a family of cysteine proteases, are crucial drivers of apoptotic activity and are involved in apoptotic signal transduction [20]. It hydrolyses both structural and functional proteins, eventually leading to cell death [21,22]. Two pathways lead to the activation of caspases: an extrinsic pathway associated with membrane receptors and their ligands, and an intrinsic pathway dependent on mitochondria [23]. Another important element in the mitochondria-mediated apoptotic signalling pathway is the Bcl-2 protein family, which includes pro-apoptotic factors such as Bax, Bad, and Bak, and antiapoptotic factors such as Bcl-2, Bcl-xl, and Bcl-w. Bax is involved in mitochondria-induced cell death, initiating apoptosis in almost all pathways and regulating the release of Cytc. After activation of the mitochondrial pathway and Cytc efflux into the cytoplasm, pro-caspase-9 is cleaved and activated by apoptosome formation, leading to the activation of caspase-3, and ultimately to apoptosis [24,25]. This report examined the gene and protein expression levels of the Bax, Cytc, and caspase families. The results showed that Bax levels were significantly increased after 24-OH-PD treatment. 24-OH-PD significantly increased Cytc in the cytoplasm, indicating that 24-OH-PD altered the permeability of the mitochondrial membrane. In addition, caspases (Caspase-3 and Caspase-9) were significantly upregulated, revealing that 24-OH-PD promotes the accumulation of Bax on the mitochondrial membrane, leading to the release of mitochondrial Cytc. Caspase-9 is cleaved and activated by the formation of apoptotic bodies, leading to the activation of Caspase-3. This eventually leads to apoptosis.

ROS are products of intracellular oxidative metabolism and are involved in regulating intracellular signalling closely associated with cancer [26,27]. ROS induces cell stress and damage through oxidative stress, resulting in cell death [28,29]. Therefore, approaches to promote ROS production are effective in the treatment of cancer [26]. Increased ROS production in the mitochondria promotes mPTP opening, resulting in the loss of membrane potential and Cytc release, thereby inducing apoptosis [30]. Besides, mPTP is a non-specific channel composed of the inner and outer membranes of mitochondria, and are involved in releasing substances within the mitochondria during cell death. Opening the mPTP is a critical event that causes cell death [31]. The reduction in mitochondrial membrane potential, mediated through the mitochondria-mediated apoptotic pathway, is one of the first intracellular events before the execution of apoptosis [32]. In the present study, the results indicated that ROS production was significantly increased in 24-OH-PD-treated cells. At the same time, mPTP was open, ΔΨm was reduced, and Cytc was released, further revealing that the pro-apoptotic effect of 24-OH-PD is closely linked to the mitochondria. NAC effectively blocked 24-OH-PD-induced ROS generation, cell viability and apoptosis in CCRF-CEM cells. Thus, 24-OH-PD stimulates mitochondria-mediated apoptosis through ROS-dependent pathways in these cells. Changes in ROS are upstream events in the process of apoptosis.

20 (S)-GRh2, a ginsenoside in ginseng, has gained great attention owing to its antiproliferative effects on various human cancer setups [33–36]. Therefore, 20 (S)-GRh2 was used as the intervention in the positive control group of the experiment. The molecular pathways underlying the effect of Rh2 on cancer cells mainly involve apoptosis. For example, Rh2 increased the level of the pro-apoptotic protein Bax, while reducing the level of the anti-apoptotic protein Bcl-2 by activating the P53 pathway. In addition, Rh2 increased the level of ROS, and the use of the antioxidant, NAC, significantly enhanced Rh2-induced apoptosis [37]. Rh2 induces Reh cell apoptosis by activating the mitochondrial pathway, thereby inducing the release of mitochondrial cytochrome c, and activating caspase-9 and caspase-3 in human leukaemic Reh cells [38]. As shown in Fig 3C, the apoptotic ratio of Rh2-treated CCRF-CEM cells increased significantly from 5.36% to 47.12%. As shown in Fig 5A and 5B, Caspases-3, Caspases -9 and Cytc were significantly upregulated (P < 0.05). As shown in Fig 4D, ΔΨm was significantly depolarised in CCRF-CEM cells after Rh2 treatment (P < 0.05). Our findings reveal that 20 (S)-GRh2 induces apoptosis in CCRF-CEM cells through a caspase-dependent mechanism and mitochondria-mediated apoptotic pathway, similar to the mechanism of 24-OH-PD. In contrast, the Bcl-2 family is not directly involved in mediating the mitochondrial apoptosis pathway, as opposed to that of 24-OH-PD drugs.

## 5. Conclusion

We observed that 24-OH-PD had a pro-apoptotic effect on CCRF-CEM cells *in vitro* and *in vivo*, through a mitochondria-mediated pathway, specifically by increasing intracellular ROS levels, opening the mPTP, and decreasing △Ψm. Increased mitochondrial membrane permeability leads to increased Bax expression and the release of Cytc, thereby activating the caspase family members and triggering the apoptotic cascade. 24-OH-PD could be developed as treatment of patients with T-ALL through preclinical, clinical safety, and efficacy studies. Cell signalling pathways are a complex network and multiple pathways should be at play and intersect during apoptosis and we can further study in the future.

## Supporting information

S1 Table Sample sequencing data quality analysis results. (DOCX)

S2 Table Primer sequences used in qRT-PCR. (DOCX)

S1 Raw images (PDF)

S1 File (ZIP)
