# Supplementary material for: Ginsenoside 24-OH-PD from red ginseng inhibits acute T-lymphocytic leukaemia by activating the mitochondrial pathway
Source: PLoS One. 2023 May 19;18(5):e0285966. doi: 10.1371/journal.pone.0285966 (PMC10198485; doi:10.1371/journal.pone.0285966)
Supplement: S1 Table — (DOCX) [file pone.0285966.s001.docx]

Supplementary table 1

Sample sequencing data quality analysis results

| Sample | Raw reads | Clean reads | Clean bases | Error rate(%) | Q30 (%) | GC content(%) |
| --- | --- | --- | --- | --- | --- | --- |
| Rh2 | 40657240 | 39849728 | 5.98G | 0.03 | 92.54 | 49.2 |
| 24 OH PD L | 42609198 | 41563186 | 6.23G | 0.03 | 92.23 | 19.8 |
| 24 OH PD M | 45575316 | 44564792 | 6.68G | 0.03 | 92.44 | 50 |
| 24 OH PD H | 45547430 | 44409180 | 6.66G | 0.03 | 92.16 | 49.7 |
| Control | 45384036 | 44303914 | 6.65G | 0.03 | 92.25 | 48.65 |
