# Supplementary material for: Ginsenoside 24-OH-PD from red ginseng inhibits acute T-lymphocytic leukaemia by activating the mitochondrial pathway
Source: PLoS One. 2023 May 19;18(5):e0285966. doi: 10.1371/journal.pone.0285966 (PMC10198485; doi:10.1371/journal.pone.0285966)
Supplement: S2 Table — (DOCX) [file pone.0285966.s002.docx]

Supplementary table 2

Primer sequences used in qRT-PCR

| Gene name | Forward primer | Reverse primer |
| --- | --- | --- |
| Bax | ATGCGTCCACCAAGAAGCTG | TGTCCACGGCGGCAATC |
| Caspase-3 | GTGGAGGCCGACTTCTTGTATGC | TGGCACAAAGCGACTGGATGAAC |
| Caspase-9 | GACCAGAGATTCGCAAACCAGAGG | AAGAGCACCGACATCACCAAATCC |
