# Supplementary material for: Ginsenoside 24-OH-PD from red ginseng inhibits acute T-lymphocytic leukaemia by activating the mitochondrial pathway
Source: PLoS One. 2023 May 19;18(5):e0285966. doi: 10.1371/journal.pone.0285966 (PMC10198485; doi:10.1371/journal.pone.0285966)
Supplement: S1 Raw images — (PDF) [file pone.0285966.s003.pdf]

The 24-OH-PD concentration sequence of all image is 0 µM, 80µM, 70µM, 60µM and 70µM Rh2.
Figure 5B is calculated from the original image by Iamge J.
NO.1 β-Actin

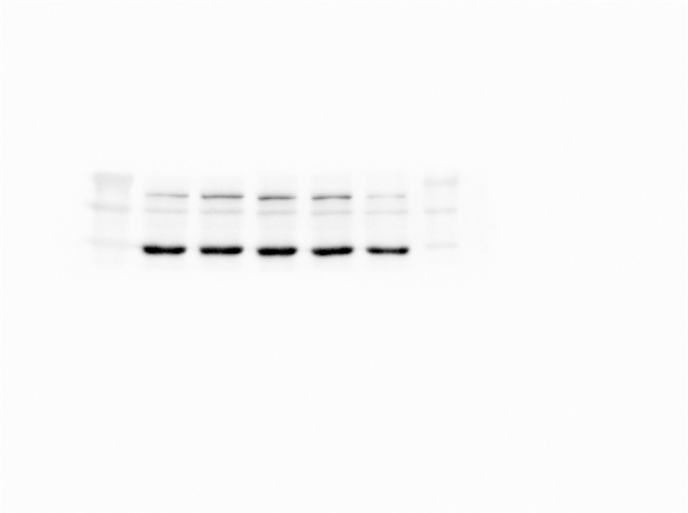

55786.279
53229.451
53270.794
52183.844
40815.744

NO.2 β-Actin

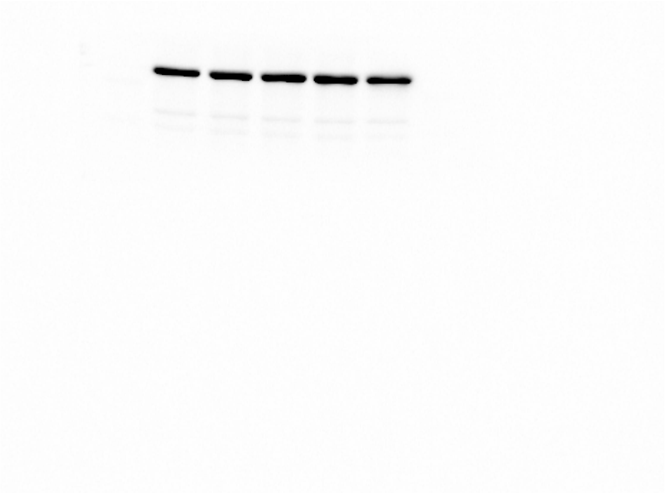

57142.38
55667.309
62115.238
62858.794
56006.966

NO.3 β-Actin

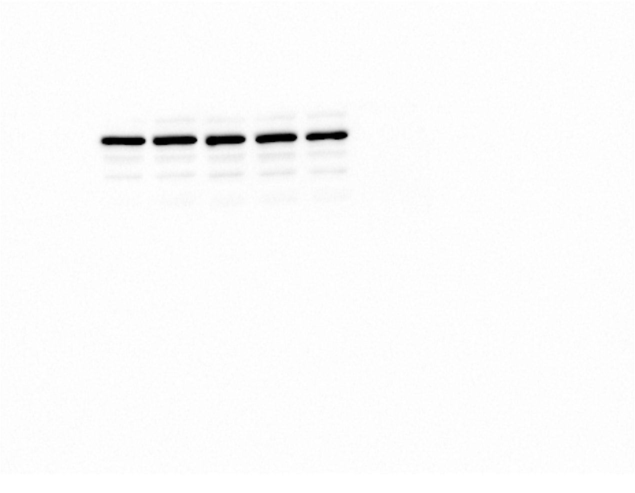

56915.459
59867.217
56566.48
57444.53
51282.551

NO.1 Cytc

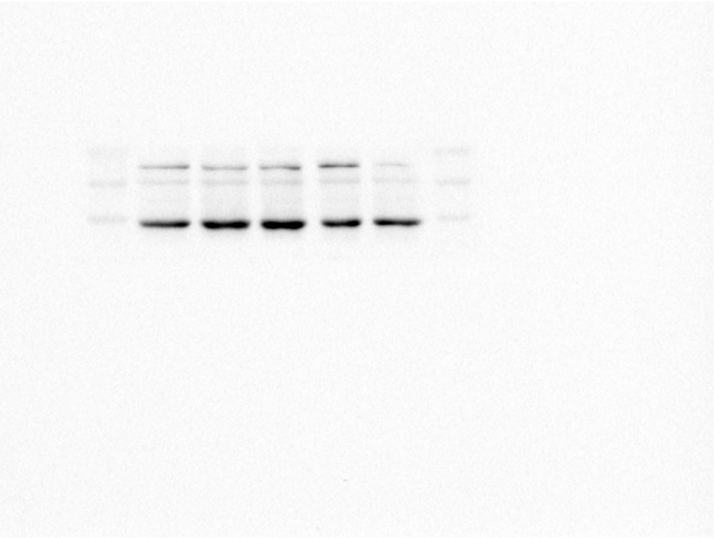

42215.158
63012.936
64651.815
50214.167
48910.037

NO.2 Cytc

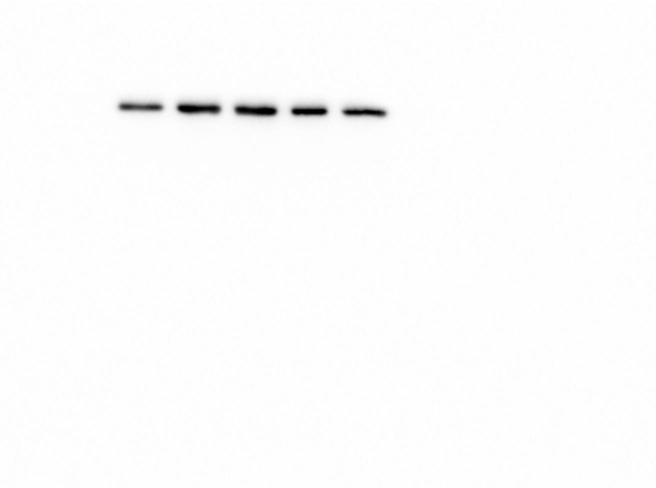

39153.087
55011.108
56537.966
37691.309
37862.865

NO.3 Cytc

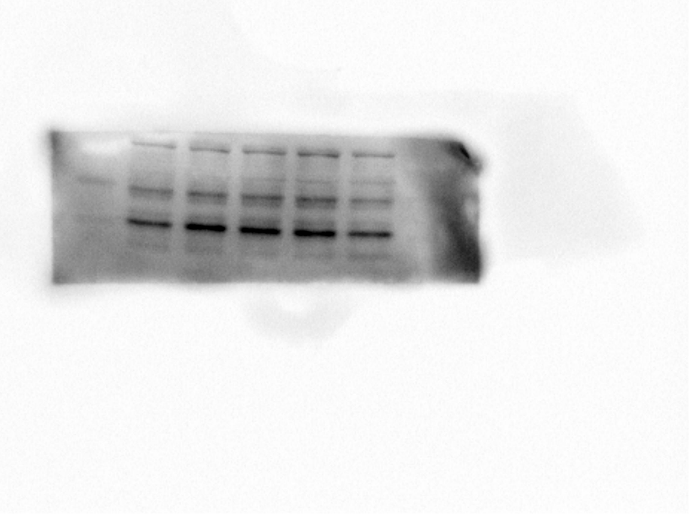

36850.229
50169.451
50843.794
52753.037
37473.622

NO.1 Caspase-3

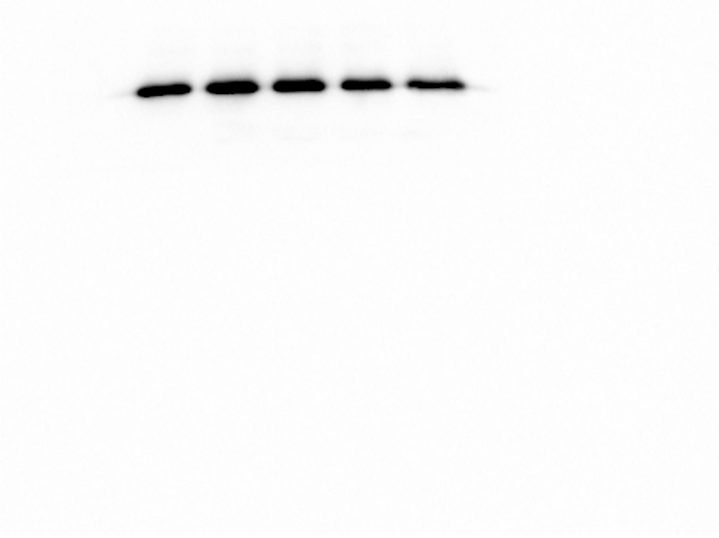

46884.723
60328.501
61267.773
62986.258
58142.329

NO.2 Caspase-3

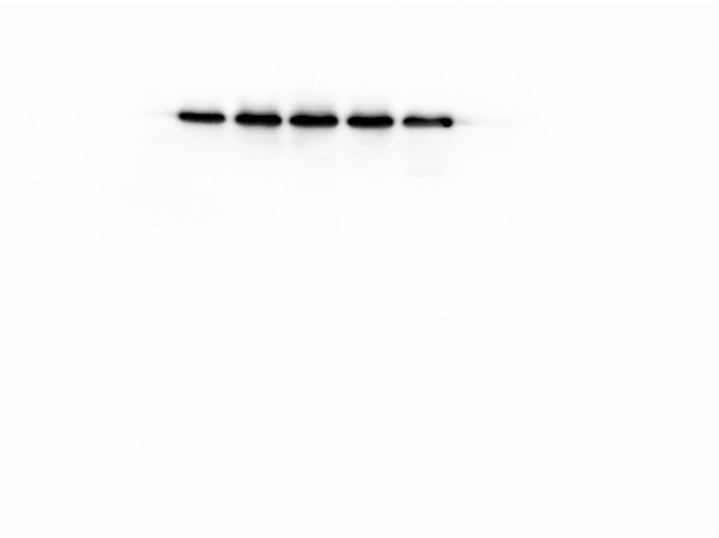

46662.43
59990.208
63590.602
57515.459
46070.572

NO.3 Caspase-3

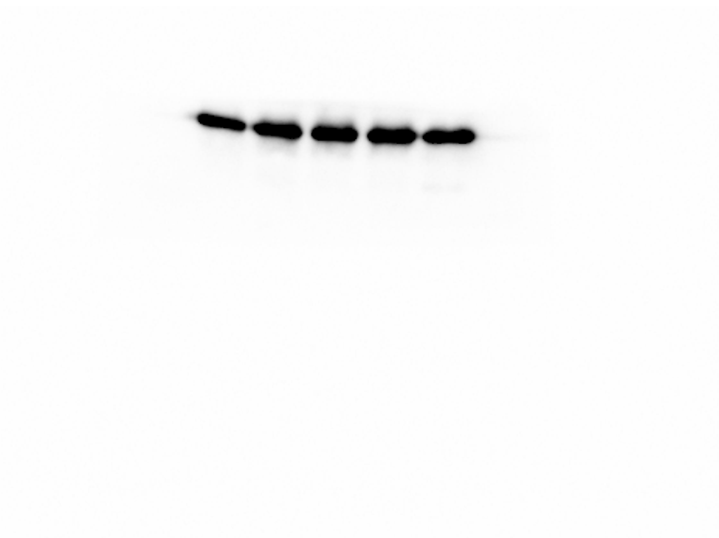

71061.693
81522.108
75537.673
61427.844
56051.472

NO.1 Caspase-9

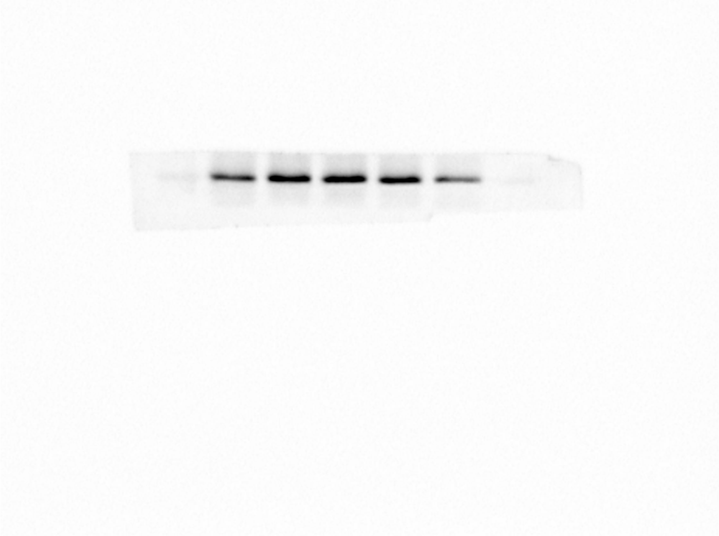

29014.208
48217.673
45648.844
44426.451
21529.823

NO.2 Caspase-9

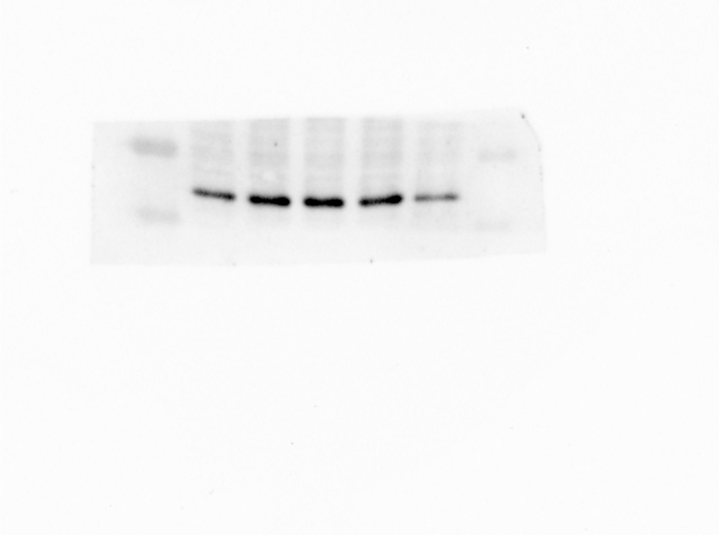

36661.501
52183.844
54289.894
48323.966
26697.43

NO.3 Caspase-9

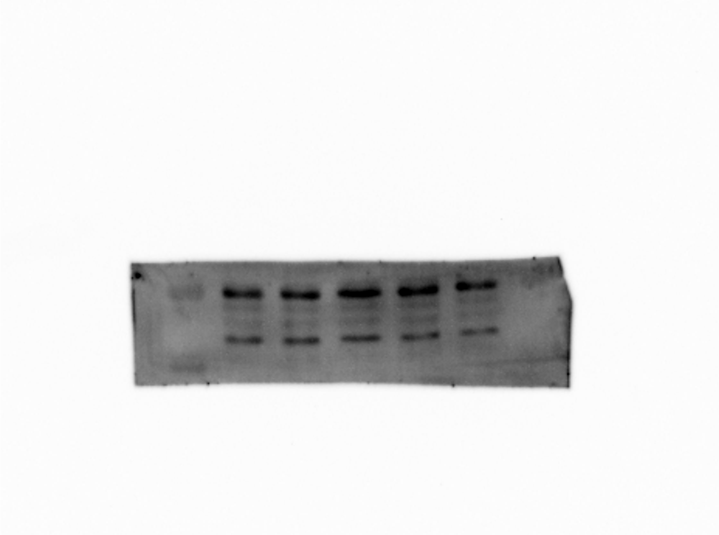

39710.966
44346.693
44995.593
41420.108
36903.744

NO.1 Bax

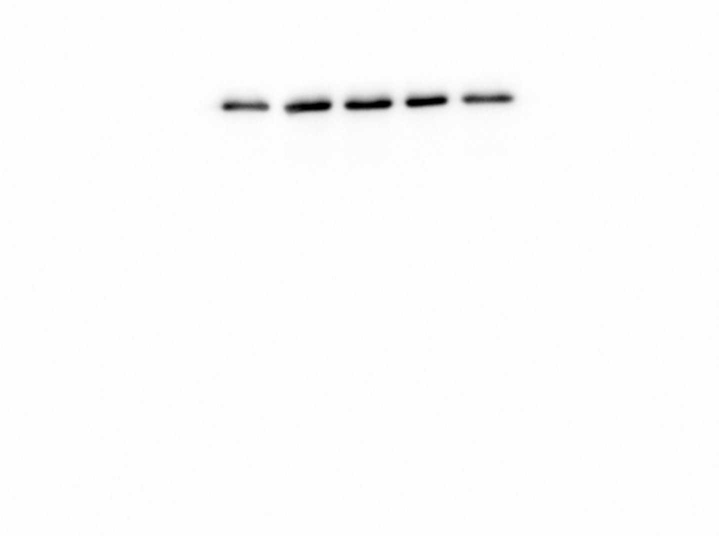

49979.886
73881.714
67934.765
65480.643
54557.25

NO.2 Bax

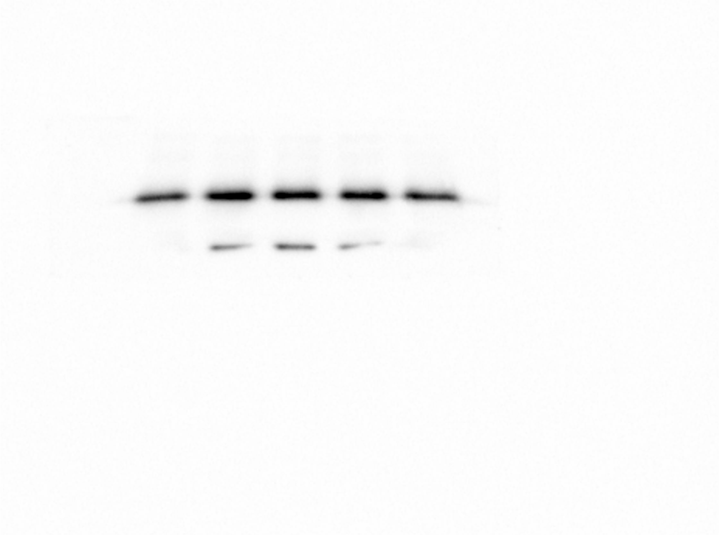

34977.057
49741.158
57249.329
53087.329
46518.179

NO.3 Bax

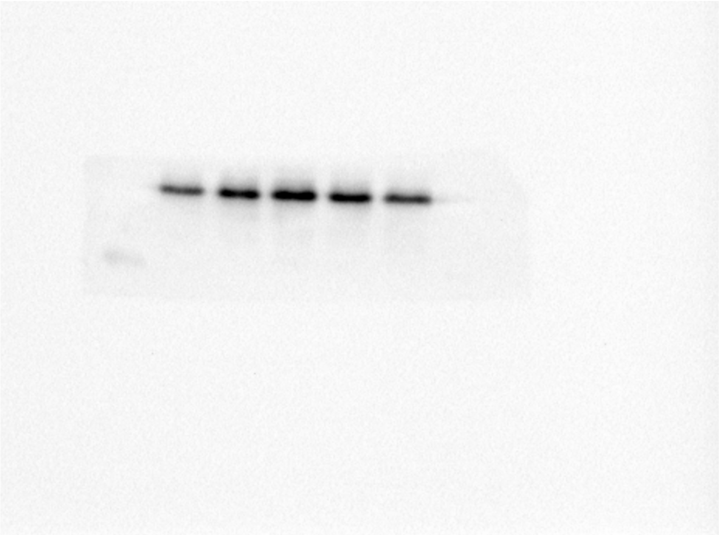

41811.744
61592.622
63581.38
54426.53
44276.401
