# Supplementary figures and images for: Ginsenoside 24-OH-PD from red ginseng inhibits acute T-lymphocytic leukaemia by activating the mitochondrial pathway
Source: PLoS One. 2023 May 19;18(5):e0285966. doi: 10.1371/journal.pone.0285966 (PMC10198485; doi:10.1371/journal.pone.0285966)

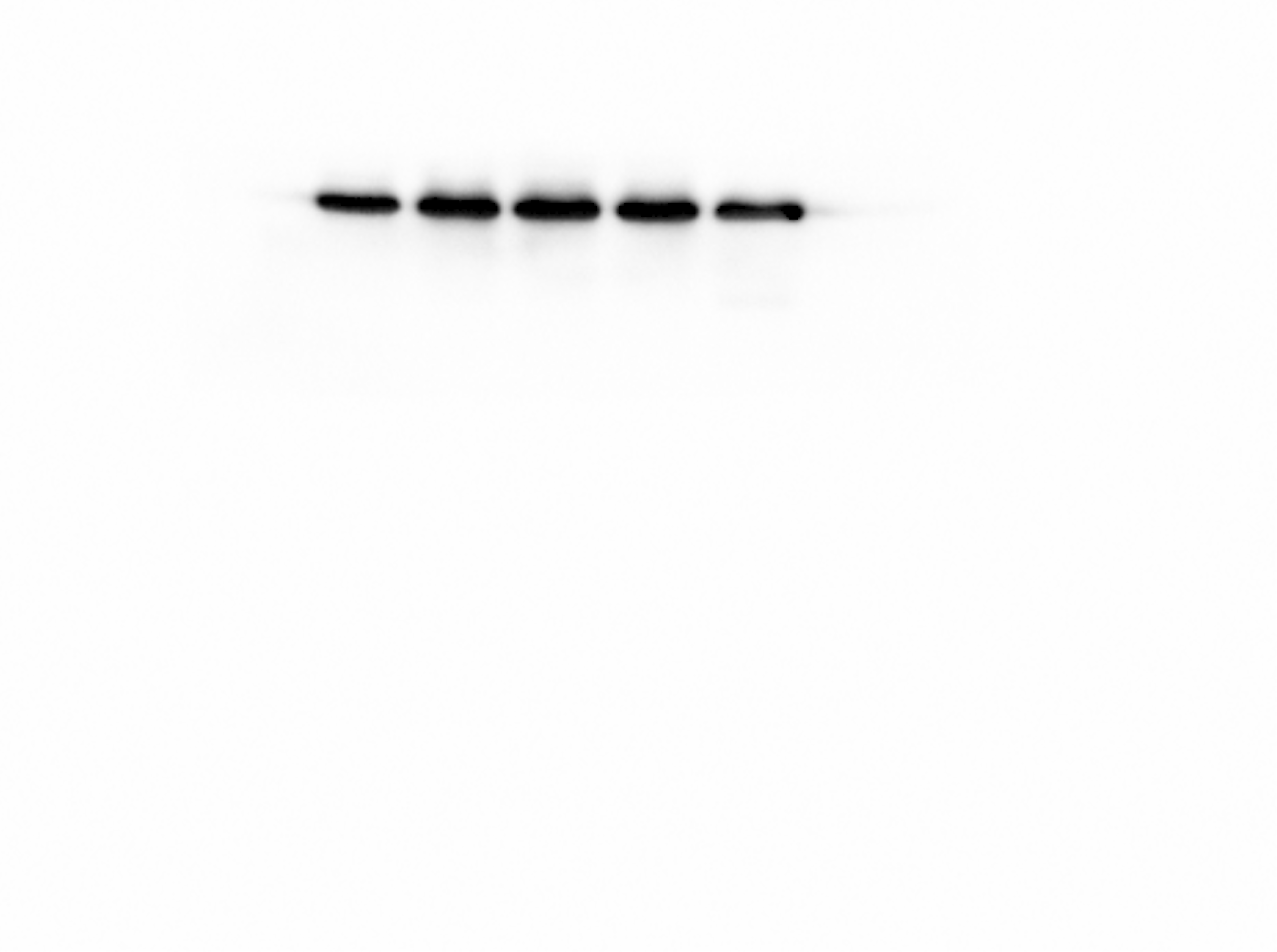

Supplement: S1 File — (ZIP) [file pone.0285966.s004.zip › wb/0 80 70 60 Rh2/NO.2 Caspase-3.tif]

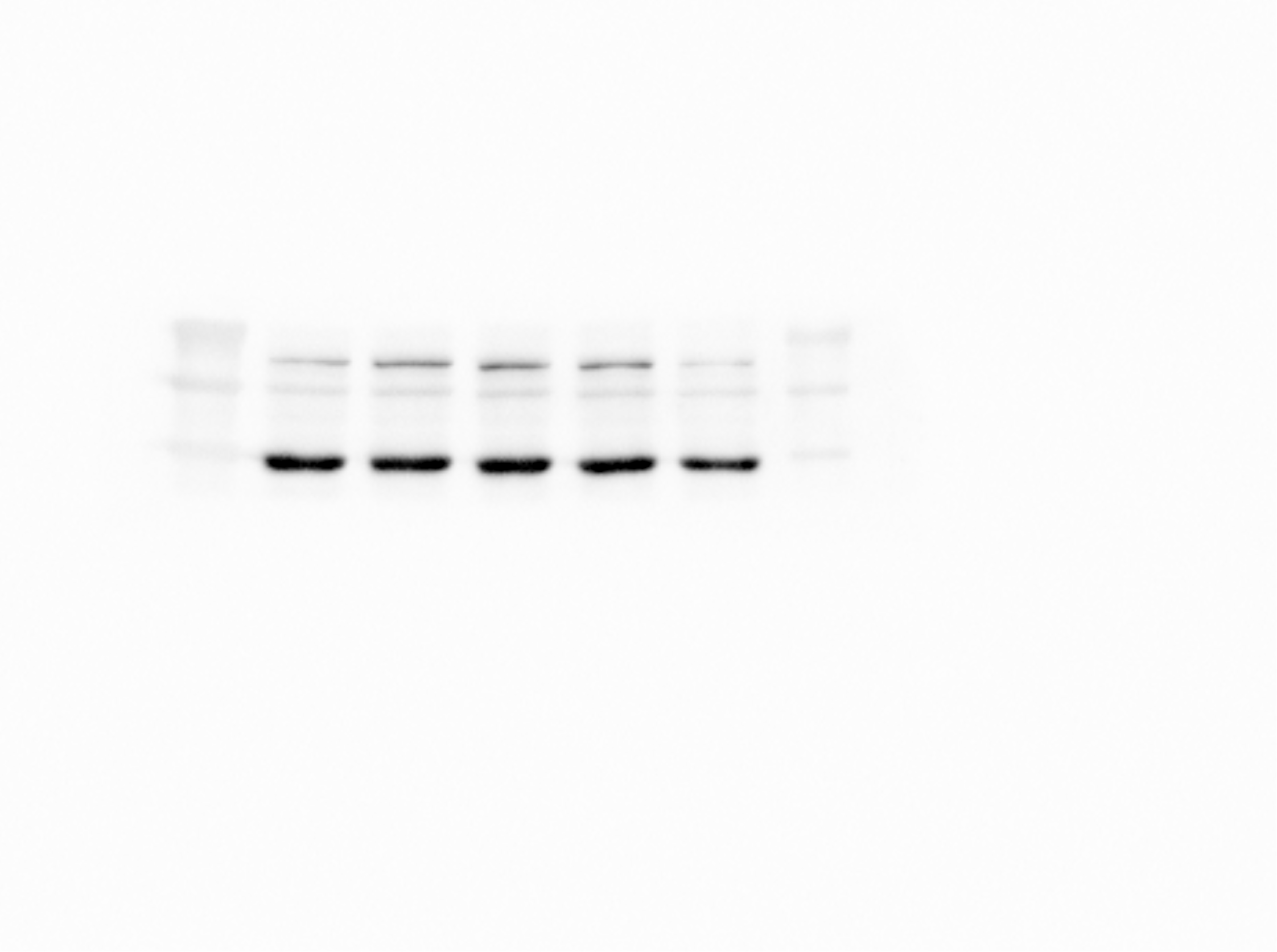

Supplement: S1 File — (ZIP) [file pone.0285966.s004.zip › wb/0 80 70 60 Rh2/NO.1 ACTB.tif]

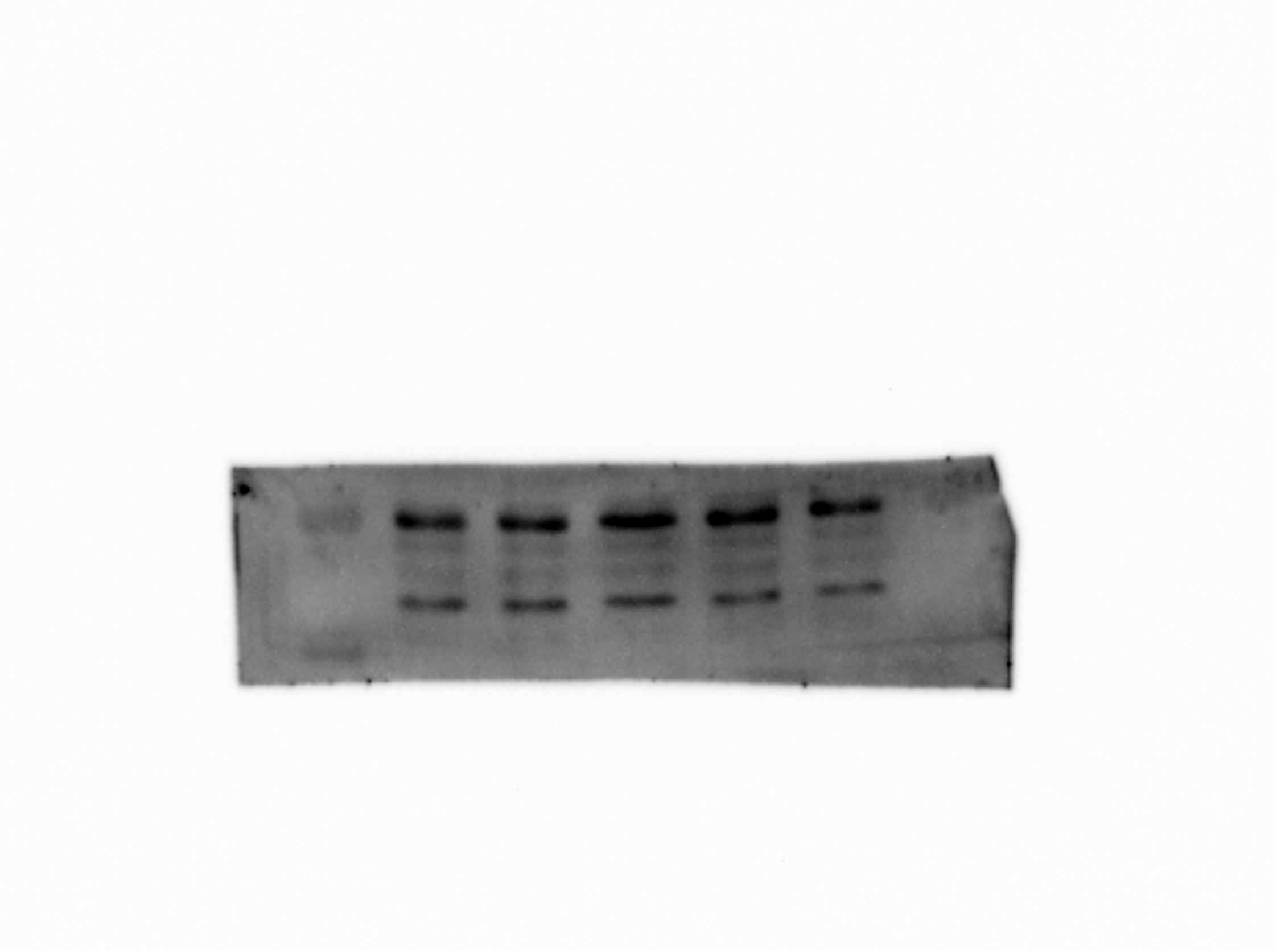

Supplement: S1 File — (ZIP) [file pone.0285966.s004.zip › wb/0 80 70 60 Rh2/NO.3 Caspase-9.tif]

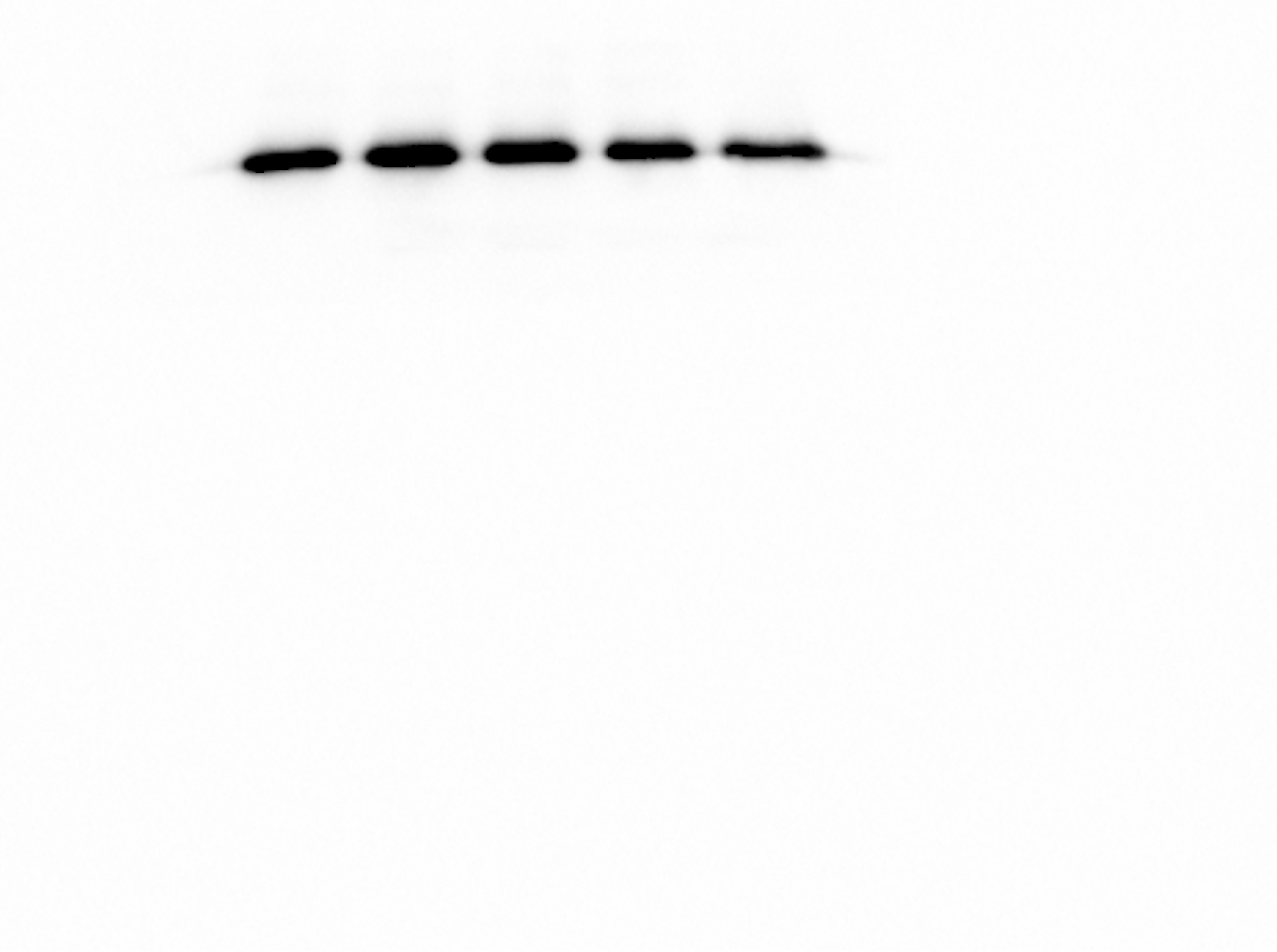

Supplement: S1 File — (ZIP) [file pone.0285966.s004.zip › wb/0 80 70 60 Rh2/NO.1 Caspase-3.tif]

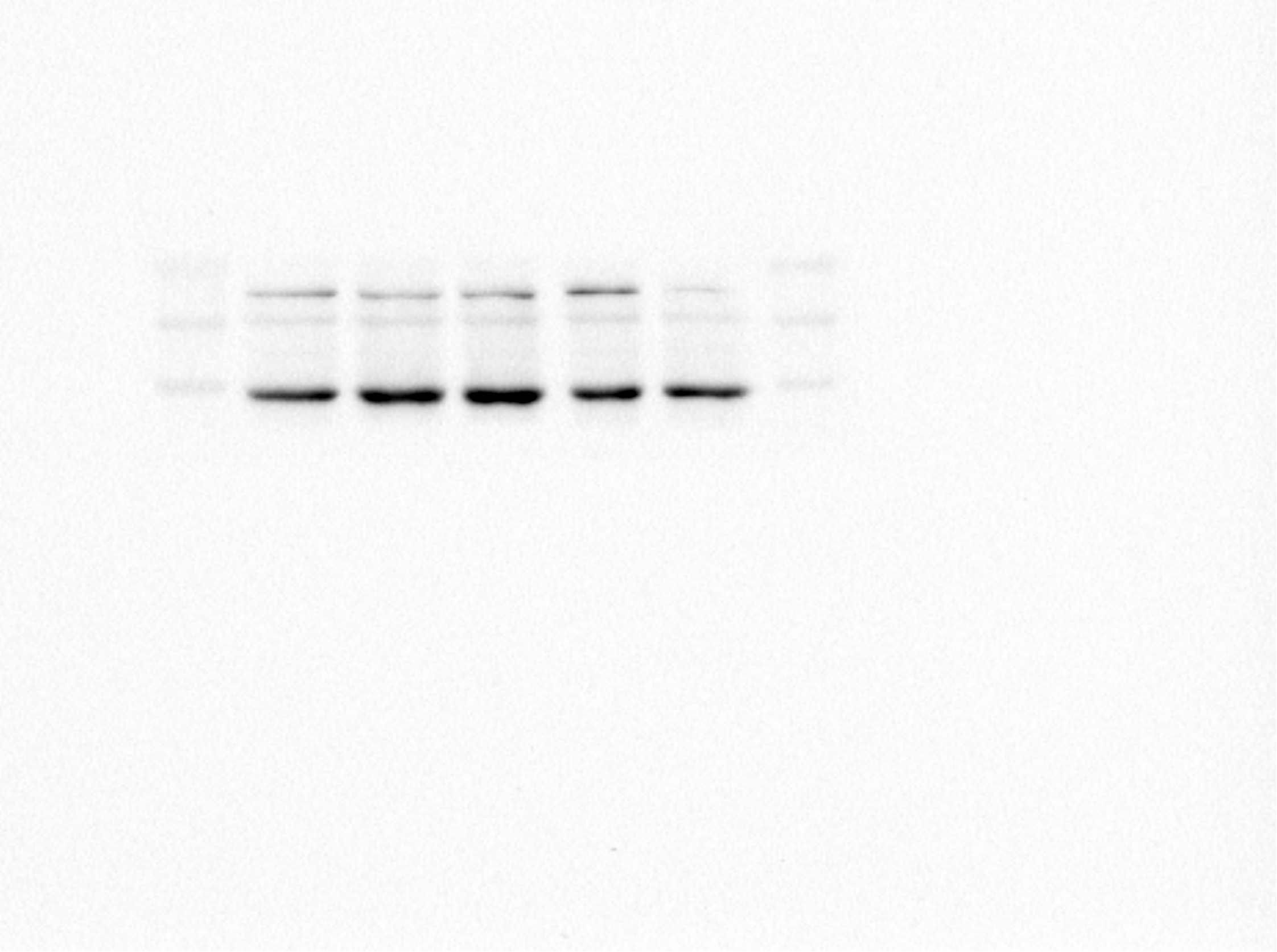

Supplement: S1 File — (ZIP) [file pone.0285966.s004.zip › wb/0 80 70 60 Rh2/NO.1 Cytc.tif]

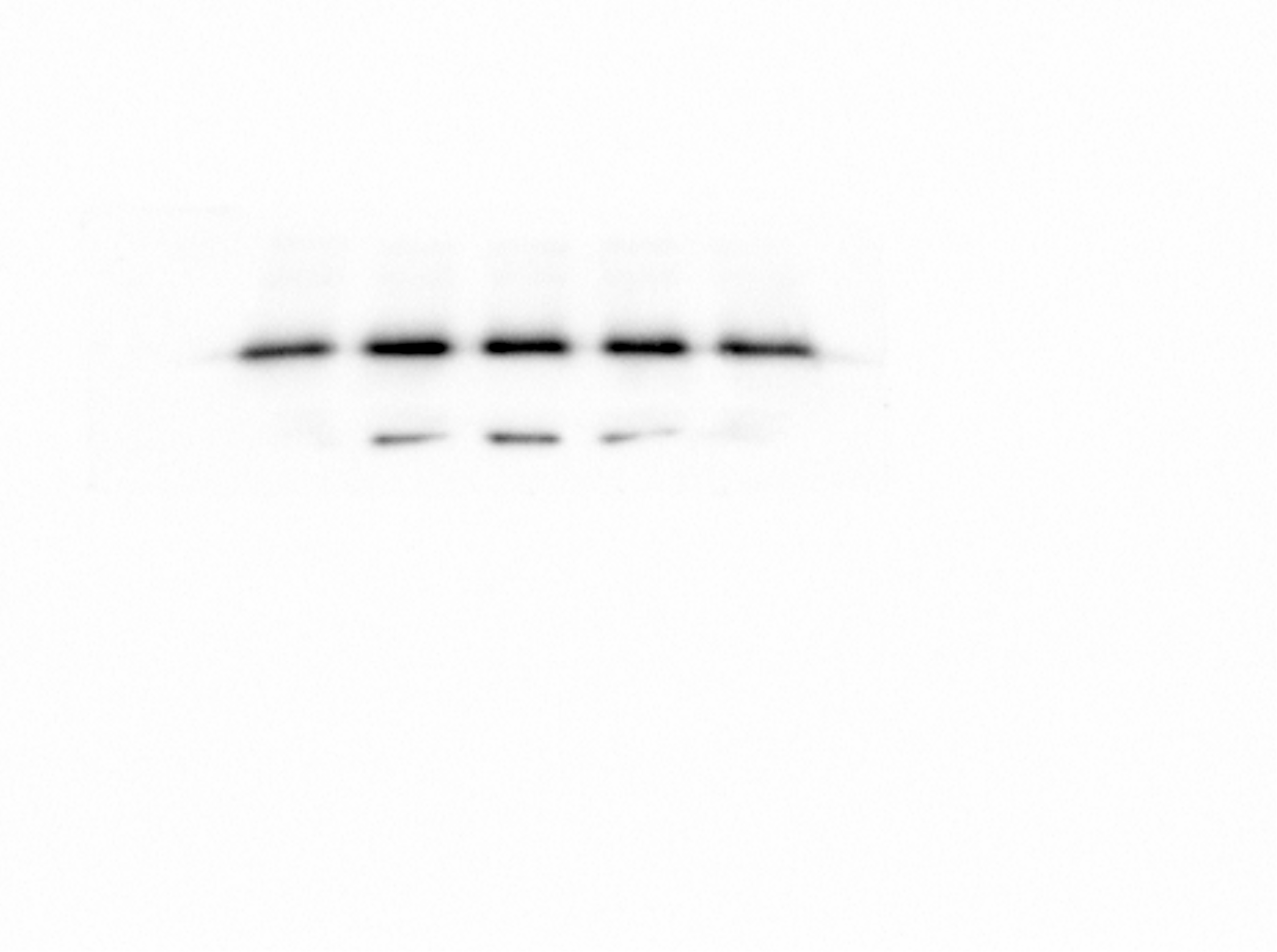

Supplement: S1 File — (ZIP) [file pone.0285966.s004.zip › wb/0 80 70 60 Rh2/NO.2 Bax.tif]

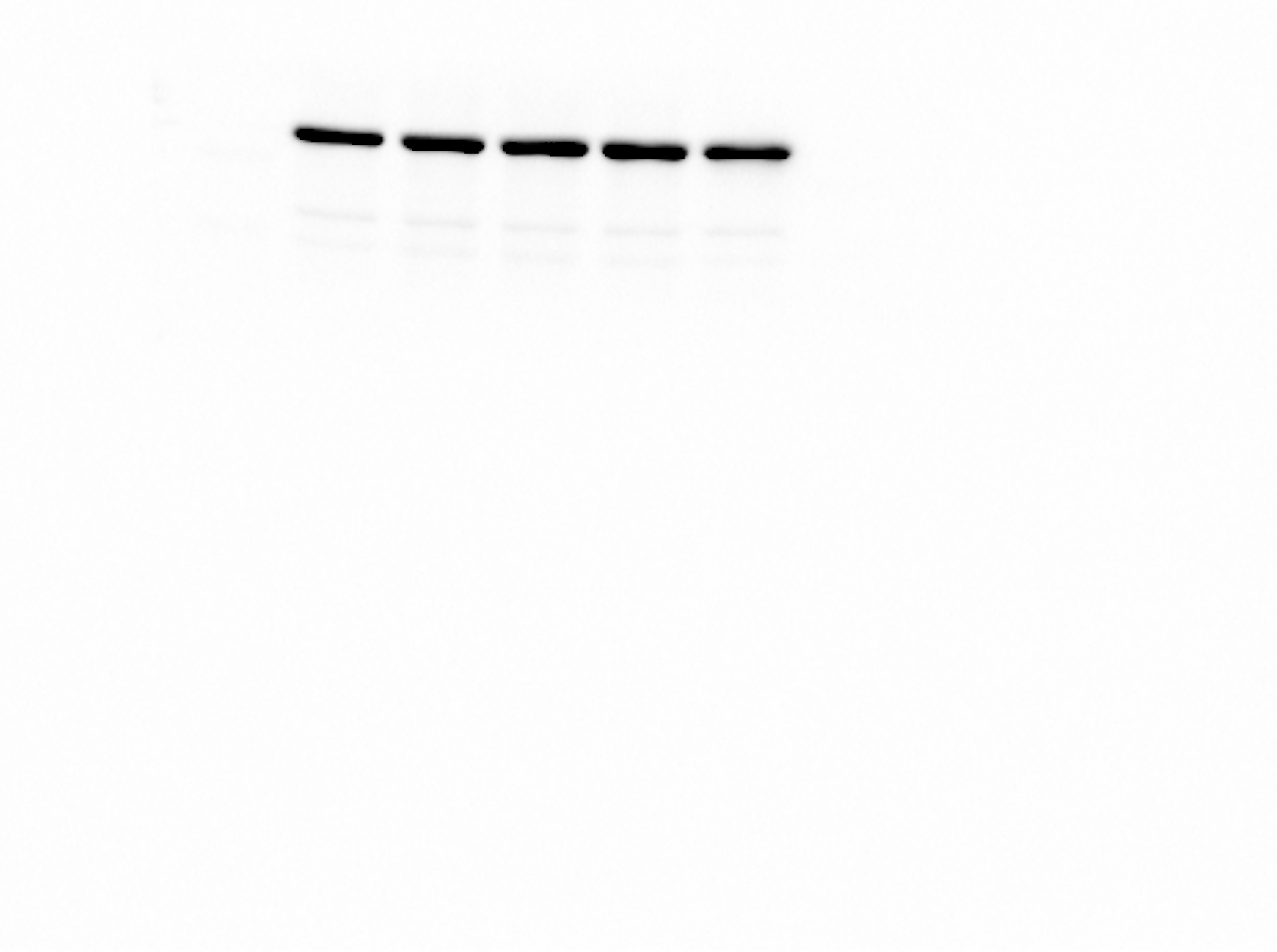

Supplement: S1 File — (ZIP) [file pone.0285966.s004.zip › wb/0 80 70 60 Rh2/NO.2 ACTB.tif]

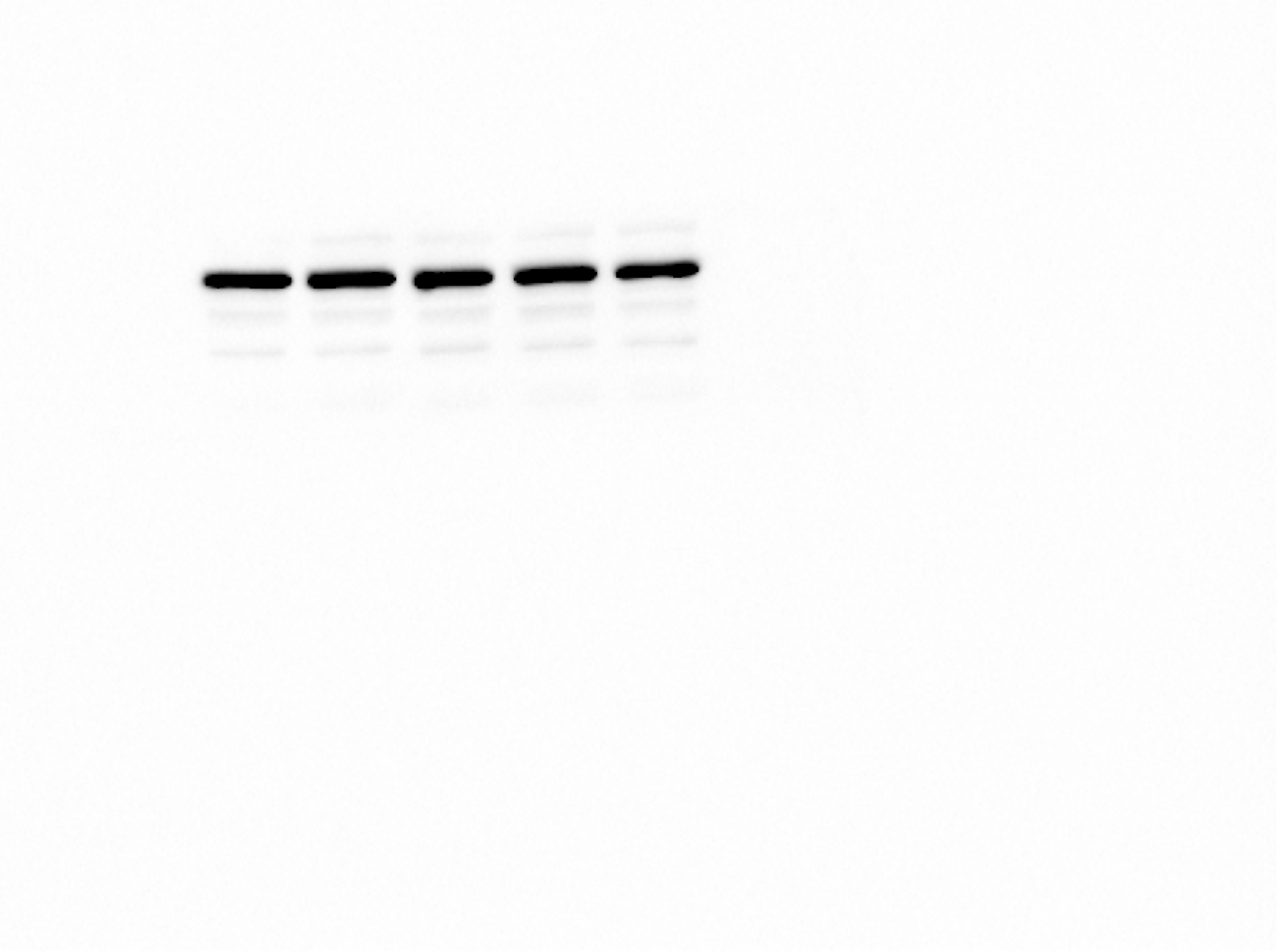

Supplement: S1 File — (ZIP) [file pone.0285966.s004.zip › wb/0 80 70 60 Rh2/NO.3 ACTB.tif]

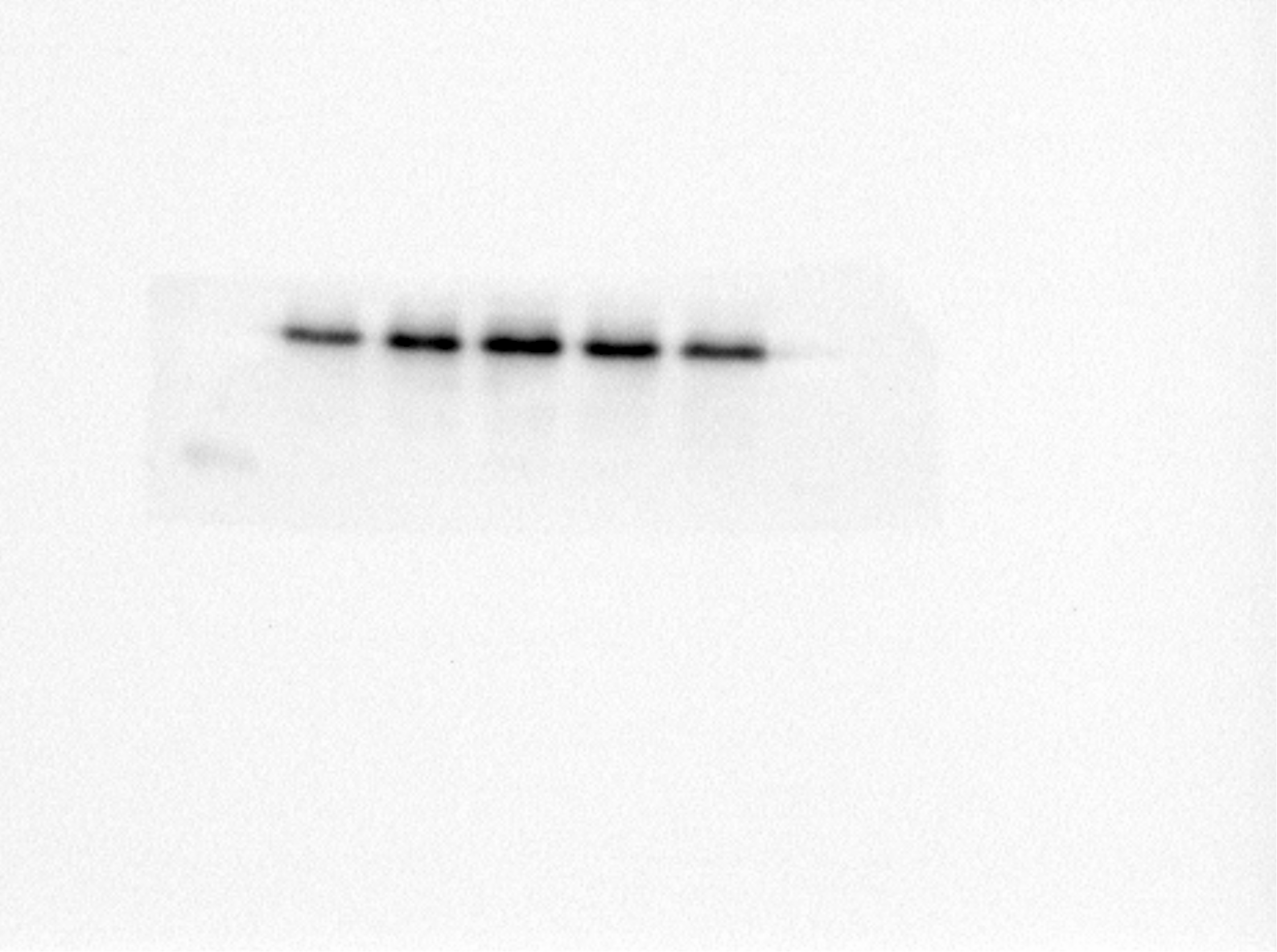

Supplement: S1 File — (ZIP) [file pone.0285966.s004.zip › wb/0 80 70 60 Rh2/NO.3 Bax.tif]

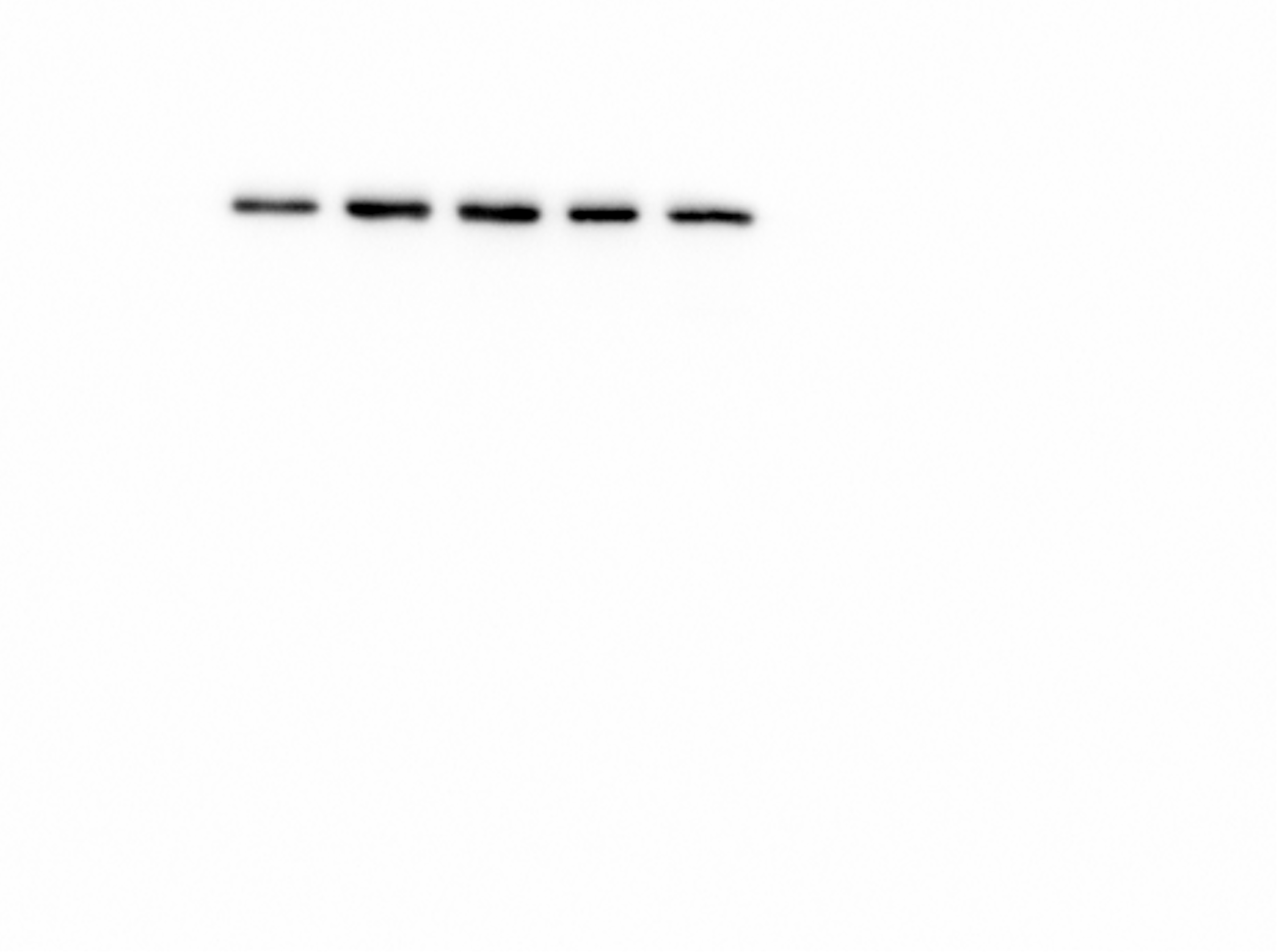

Supplement: S1 File — (ZIP) [file pone.0285966.s004.zip › wb/0 80 70 60 Rh2/NO.2 Cytc.tif]

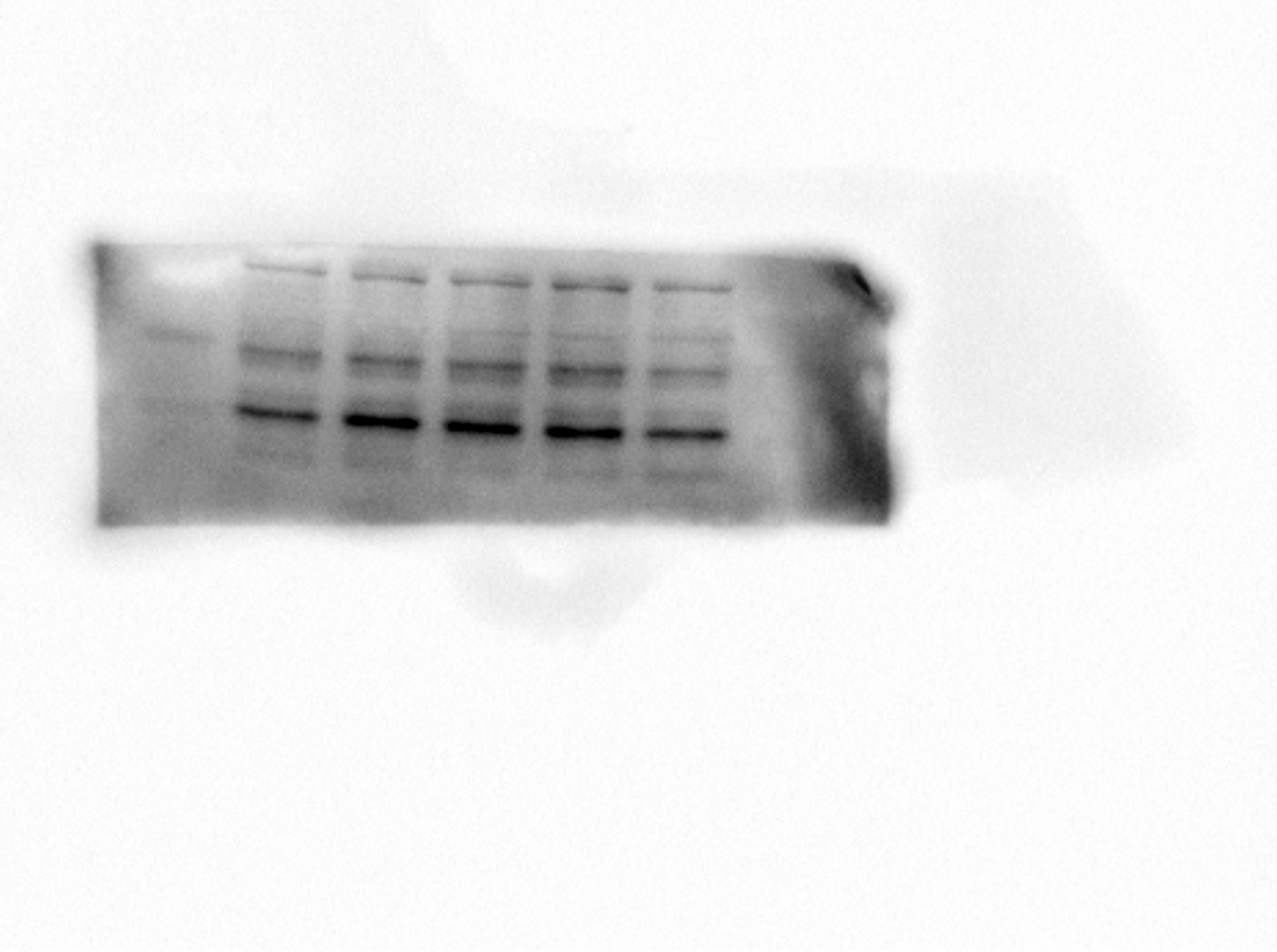

Supplement: S1 File — (ZIP) [file pone.0285966.s004.zip › wb/0 80 70 60 Rh2/NO.3 Cytc.tif]

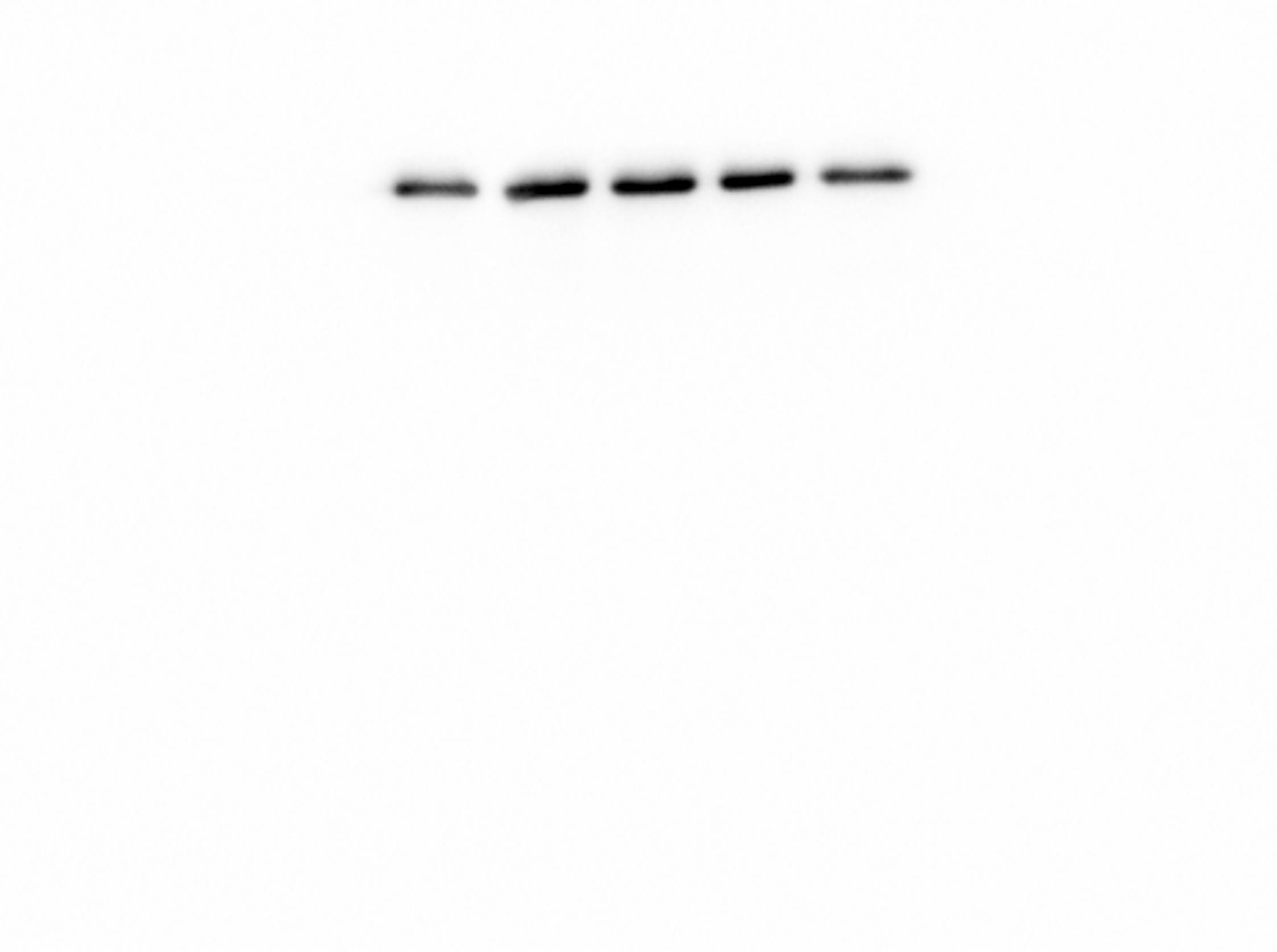

Supplement: S1 File — (ZIP) [file pone.0285966.s004.zip › wb/0 80 70 60 Rh2/NO.1 Bax.tif]

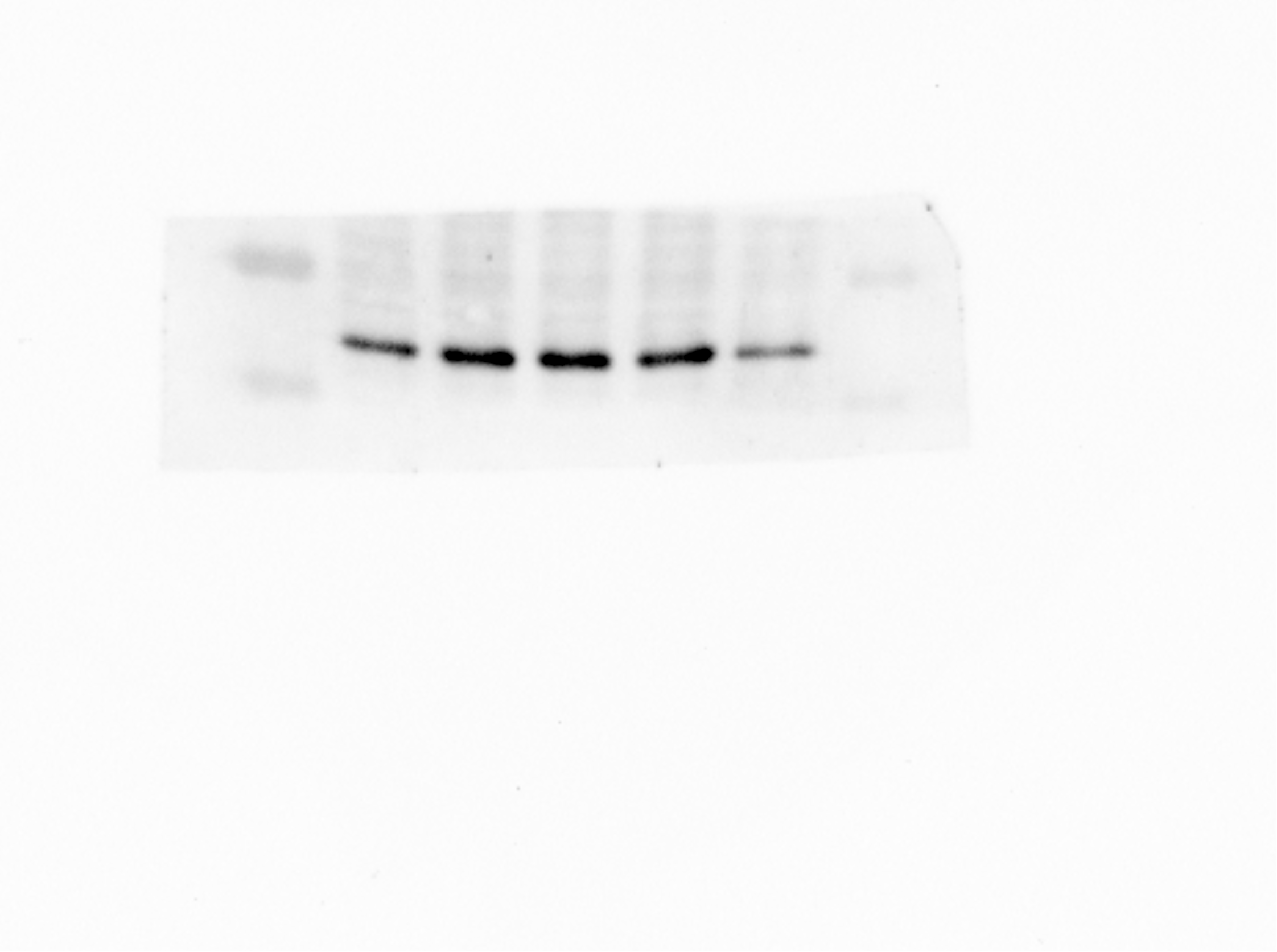

Supplement: S1 File — (ZIP) [file pone.0285966.s004.zip › wb/0 80 70 60 Rh2/NO.2 Caspase-9.tif]

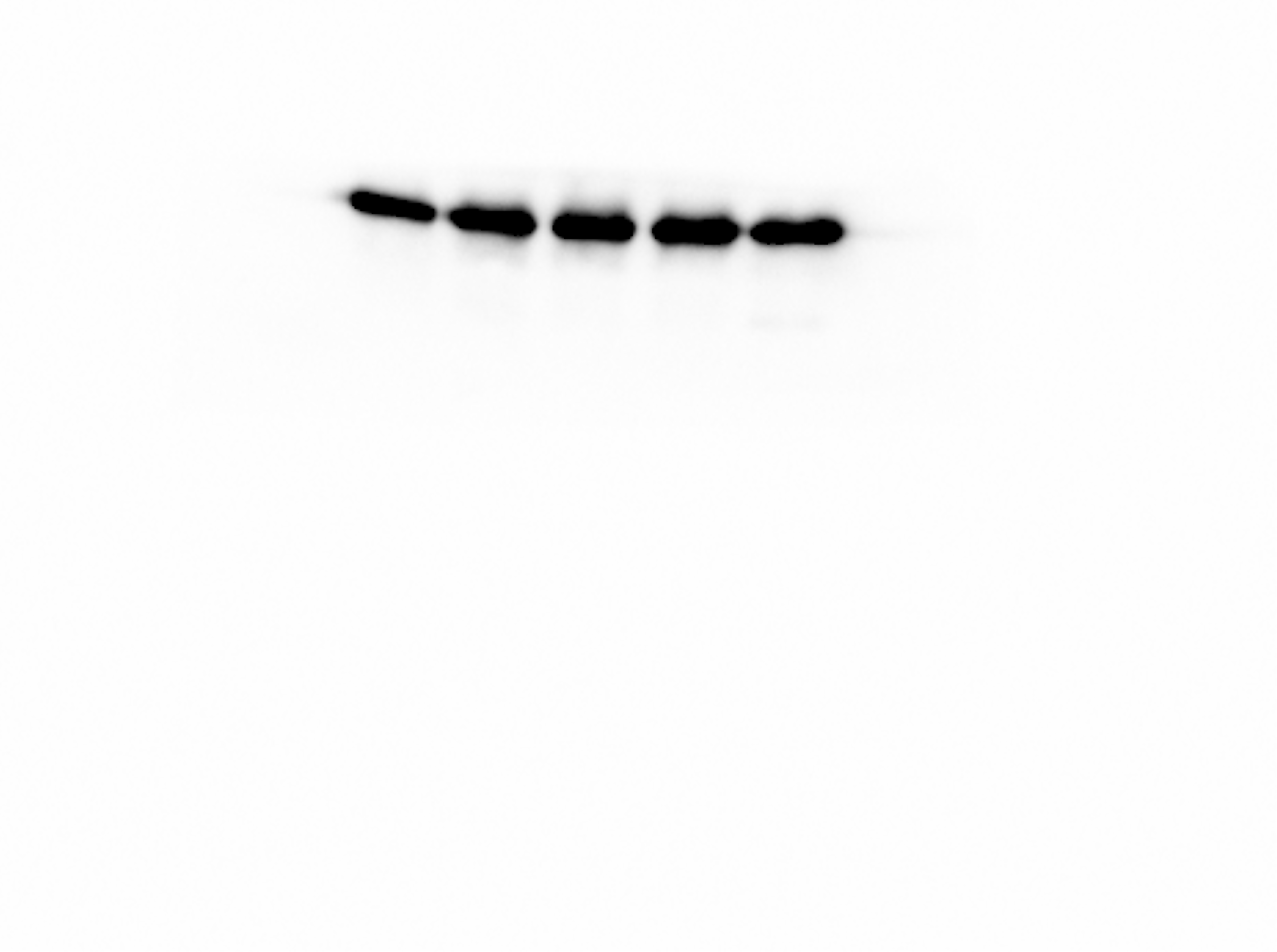

Supplement: S1 File — (ZIP) [file pone.0285966.s004.zip › wb/0 80 70 60 Rh2/NO.3 Caspase-3.tif]

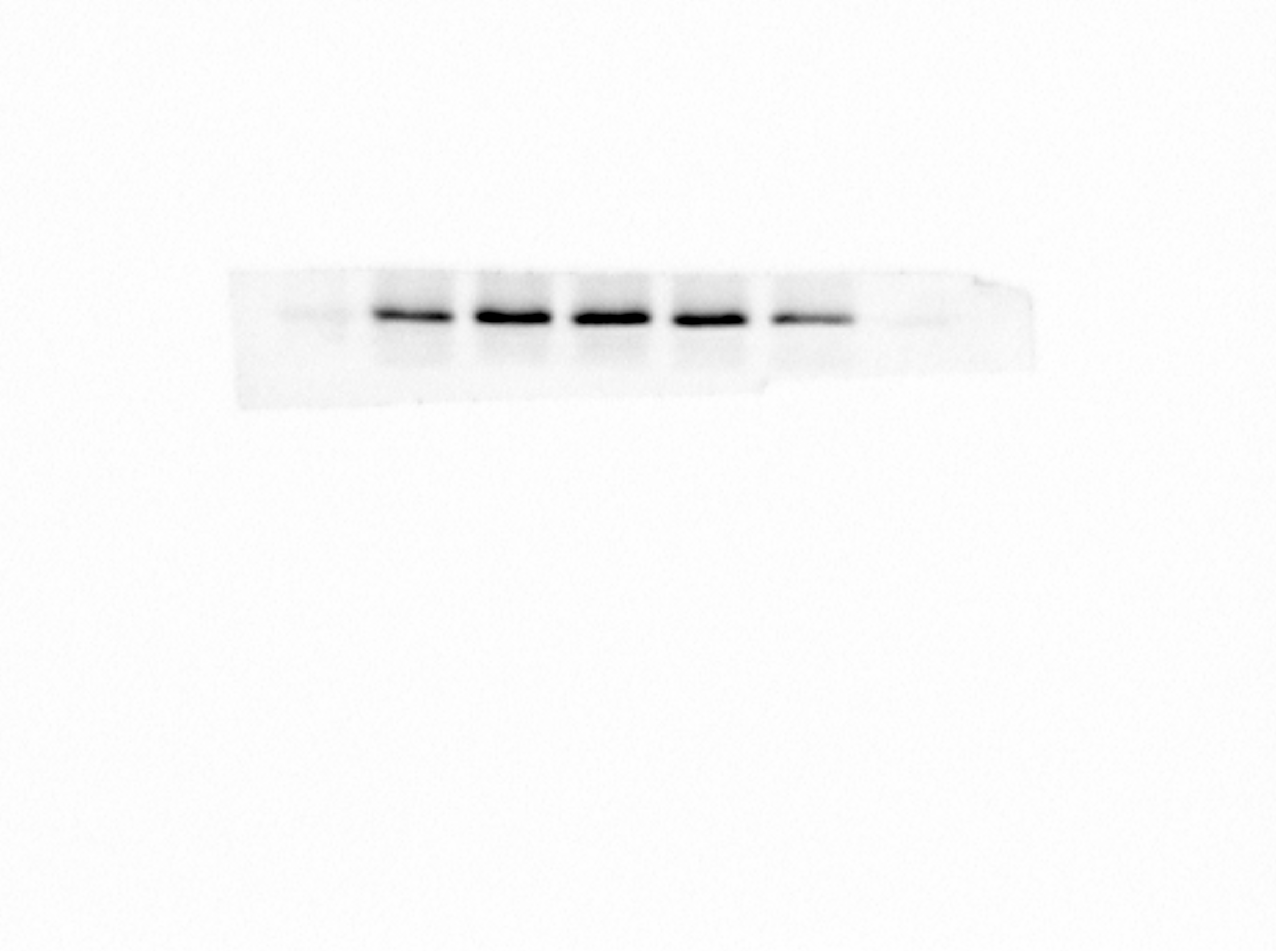

Supplement: S1 File — (ZIP) [file pone.0285966.s004.zip › wb/0 80 70 60 Rh2/NO.1 Caspase-9.tif]
